# Supplementary material for: Light-gated integrator for highlighting kinase activity in living cells
Source: Nat Commun. 2024 Sep 6;15:7804. doi: 10.1038/s41467-024-51270-4 (PMC11379911; doi:10.1038/s41467-024-51270-4)
Supplement: Supplementary file 1 — Supporting information [file 41467_2024_51270_MOESM1_ESM.pdf]

Supplementary Figures

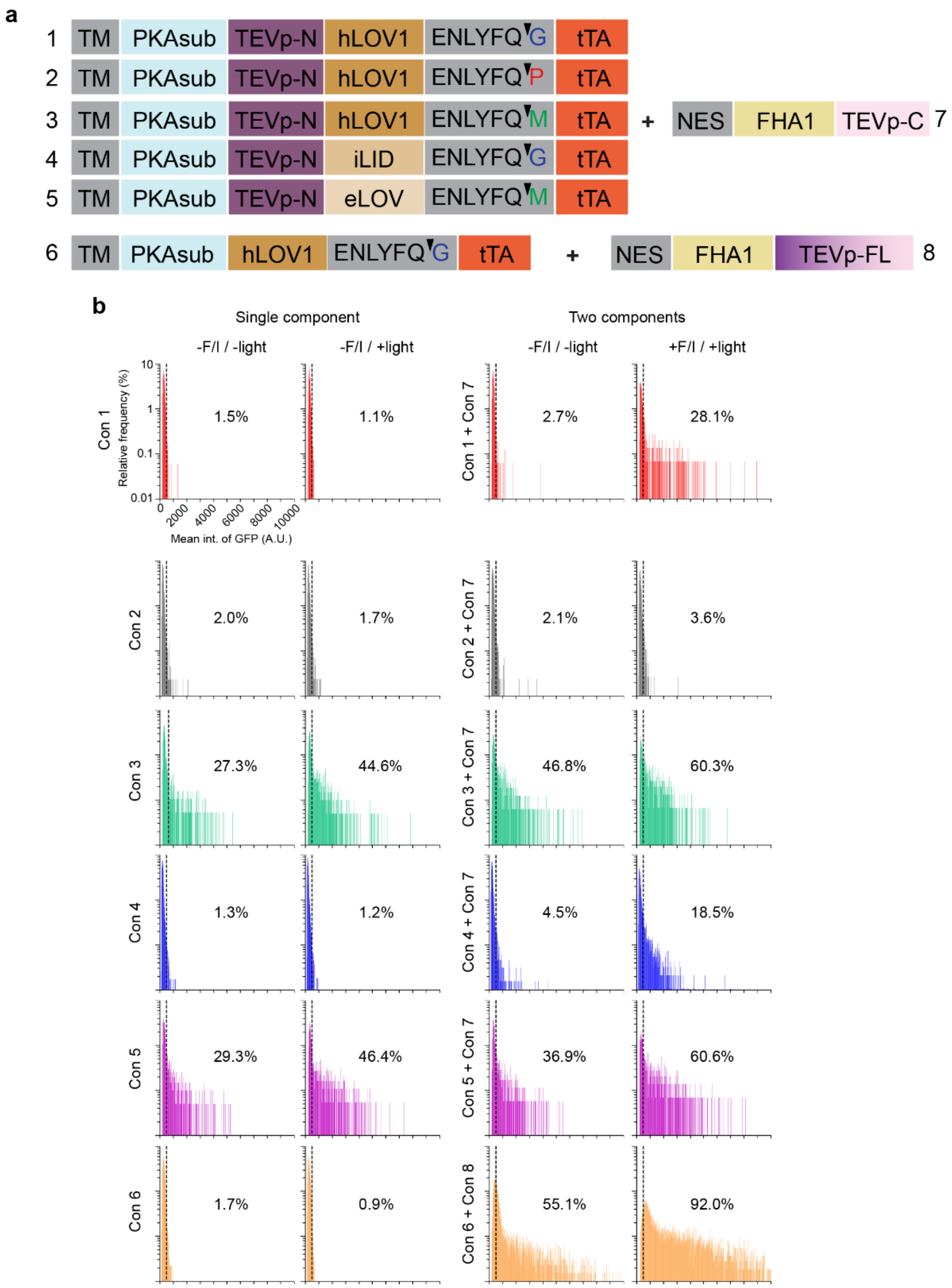

Supplementary Fig. 1 | KINACT optimization. a, Domain structures of various combinations of A-KINACT components 1 and 2. **b**, Histograms showing induced H2B-EGFP expression in HEK293T cells expressing different versions of component 1, either alone under dark or light conditions (left) or co-expressed with different versions of component 2 under untreated/dark or treated/light conditions (right). The fraction of H2B-EGFP^{+} cells above the threshold cut-off (dashed line) is indicated. Data from 3 independent experiments. Source data are provided as a Source Data file.

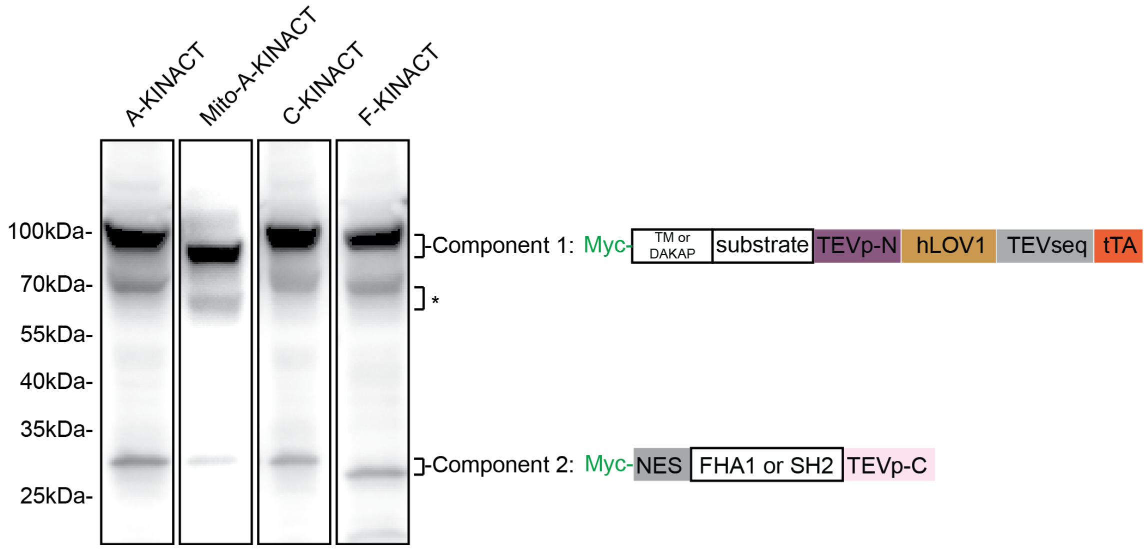

Supplementary Fig. 2 | Representative western blot characterization of main KINACT integrators expression. Bands at ~100 kDa correspond to component 1 of each KINACT, and bands between 25 kDa and 35 kDa correspond to component 2 of each KINACT. Both components 1 and 2 were fused to an N-terminal Myc epitope and blotted using an anti-Myc antibody. *Weak bands at ~70kDa correspond to truncations of component 1 that lack the tTA domain and have no transcriptional activity.

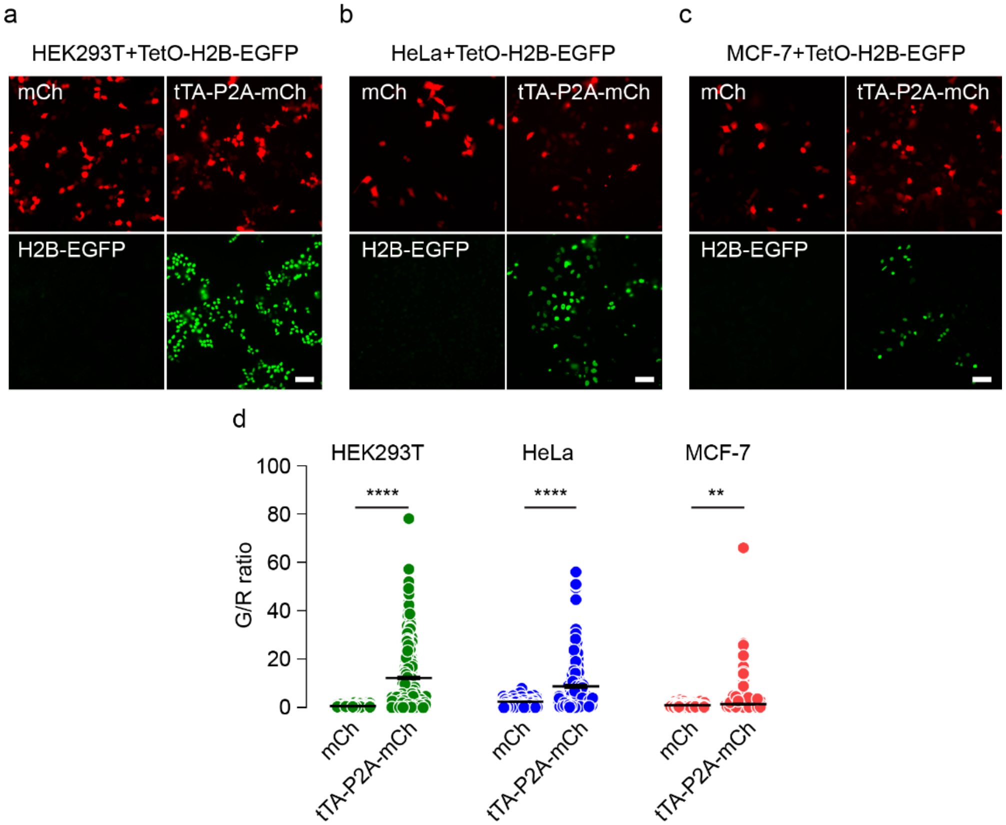

Supplementary Fig. 3 | Characterization of the ability to induce TetO-H2B-EGFP expression in stable cell lines. a-c, Representative images of H2B-EGFP expression induced by tTA transcription factor in HEK293T (a), HeLa (b) and MCF-7 (c) cells stably integrating TetO-H2B-EGFP. Scale bars, 10 μm. **d**, Quantification of G/R ratio of mCherry^{+} cells. ***P* = 0.0015, *****P* < 0.0001. Statistical analysis was performed using unpaired two-tailed Student *t*-test. Data from 3 independent experiments. Data are mean ± s.e.m. Source data are provided as a Source Data file.

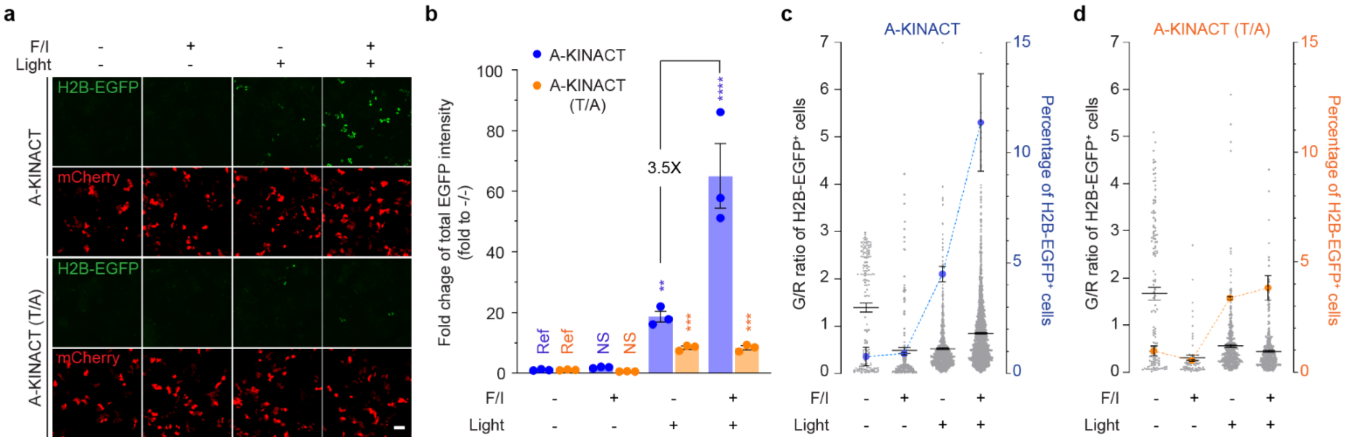

Supplementary Fig. 4 | Transient expression of A-KINACT for cumulative PKA activity in TetO-H2B-EGFP HEK293T cells. a, Snapshot imaging of cumulative PKA activity in live cells. Four treatment conditions were applied: -F/I/-light, +F/I/-light, -F/I/+light and +F/I/+light. Scale bar, 10 µm. **b**, Statistical quantification of total EGFP intensity under all conditions. $^{**}P = 0.0011$ (A-KINACT, -F/I/+light), $^{****}P < 0.0001$ (A-KINACT, +F/I/+light), $^{***}P = 0.0002$ (T/A, -F/I/+light) and $^{***}P = 0.0002$ (T/A, +F/I/+light). Statistical analysis was performed using ordinary one-way ANOVA followed by Dunnett's multiple-comparisons test. NS, not significant. **c**, The fraction and G/R ratio of H2B-EGFP$^+$ cells expressing A-KINACT. n = 143, n = 183, n = 836 and n = 2081 cells. **d**, The fraction and G/R ratio of H2B-EGFP$^+$ cells expressing A-KINACT (T/A). n = 133, n = 67, n = 442 and n = 452 cells. For **b-d**, data from 3 independent experiments. Data are mean ± s.e.m. Source data are provided as a Source Data file.

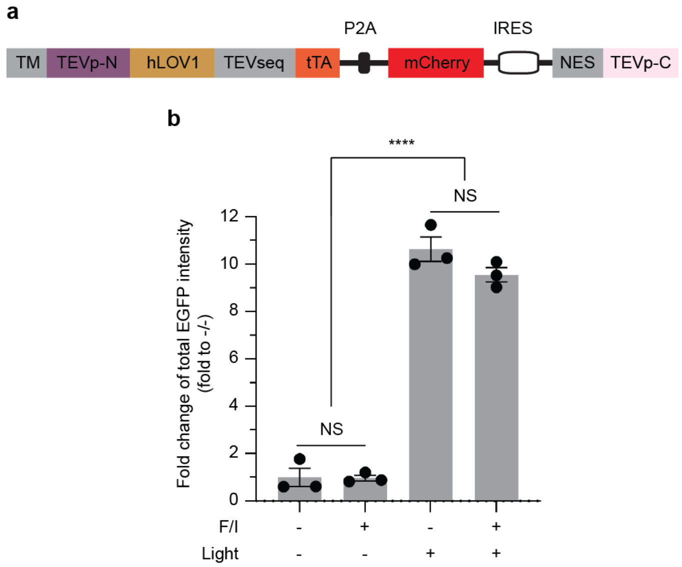

Supplementary Fig. 5 | Overexpression of TEV-only integrator in HEK293T cells indicating spontaneous binding of components 1 and 2. a, Domain structure of TEV-only integrator. The PKA substrate peptide and FHA1 domain were removed from A-KINACT. **b**, Statistical quantification of total EGFP intensity under -F/I/-light, +F/I/-light, -F/I/+light and +F/I/+light conditions. ****$P < 0.0001$. Statistical analysis was performed using ordinary one-way ANOVA followed by Tukey's multiple-comparisons test. NS, not significant. Data from 3 independent experiments. Data are mean ± s.e.m. Source data are provided as a Source Data file.

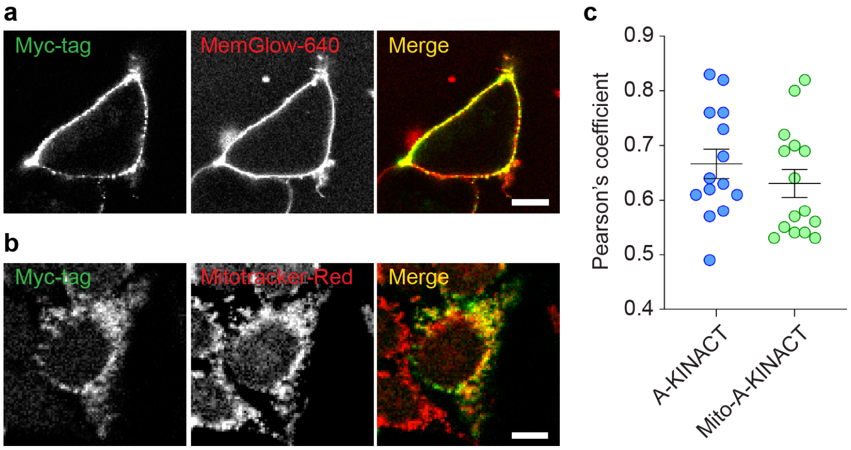

Supplementary Fig. 6 | Colocalization of A-KINACT and Mito-A-KINACT with corresponding organelle markers. **a**, Representative images of A-KINACT (green channel, Myc-tag immunostaining), MemGlow-640 (far red channel, plasma membrane dye), and merged image. **b**, Representative images of Mito-A-KINACT (green channel, Myc-tag immunostaining), Mitotracker-Red (red channel, mitochondria dye) and merged image. Scale bars, 10 µm. **c**, Quantification of Pearson's coefficient of A-KINACT and MemGlow-640 (n = 14 cells) and Mito-A-KINACT and Mitotracker-Red (n = 15 cells). Data are mean ± s.e.m. Source data are provided as a Source Data file.

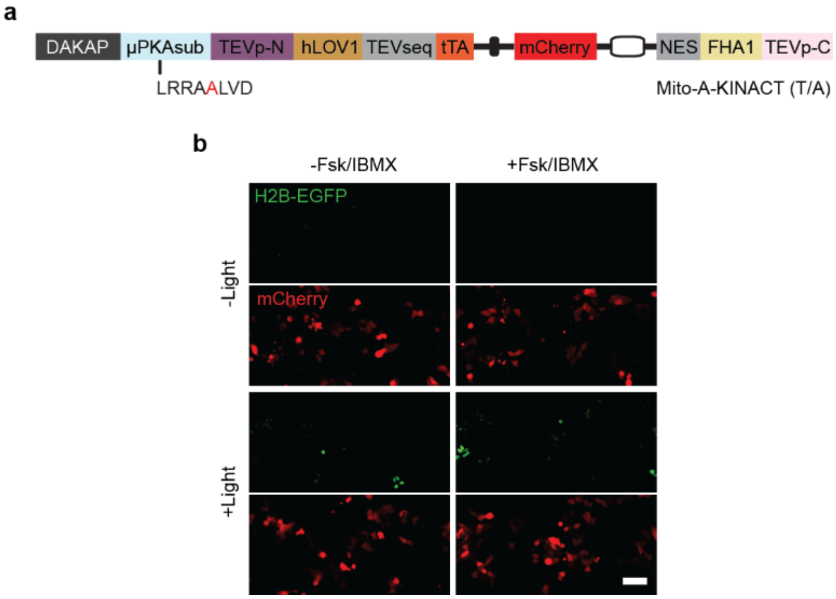

Supplementary Fig. 7 | Images of compartmentalized PKA activity using nonphosphorylatable Mito-targeted A-KINACT (T/A). a, Domain structure of outer mitochondrial membrane-targeted A-KINACT (T/A). **b**, Snapshot imaging of spontaneously induced H2B-EGFP expression. Four treatment conditions were used: -F/I/-light, +F/I/-light, -F/I/+light and +F/I/+light. Scale bar, 10 μm.

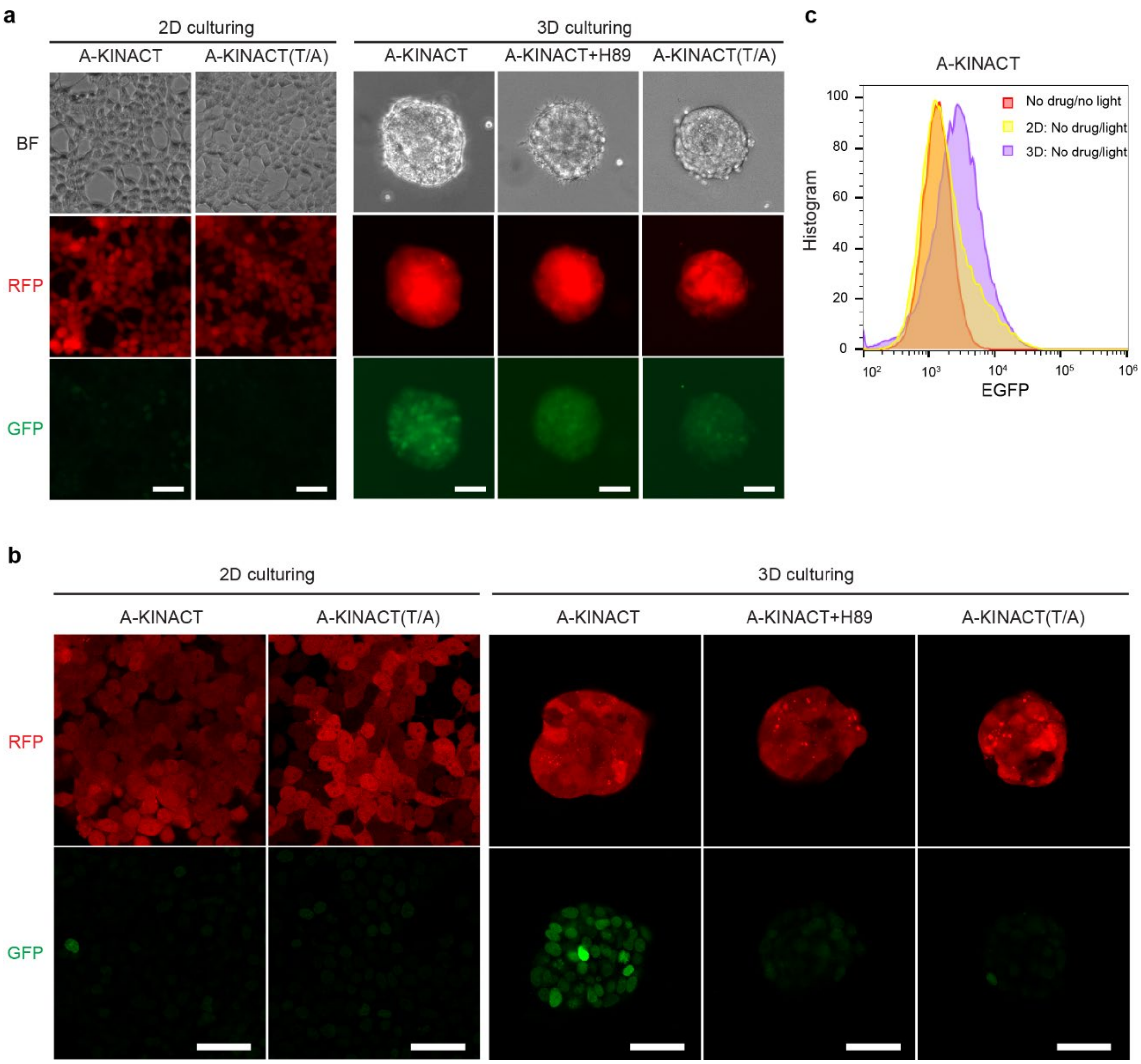

Supplementary Fig. 8 | Comparison of basal PKA activities between 2D and 3D culture conditions. a, Epifluorescence imaging of blue light-induced H2B-EGFP expression in A-KINACT and A-KINACT (T/A) dual stable cells performed on an EVOS tissue culture microscope (Invitrogen). **b**, Confocal imaging of blue light-induced H2B-EGFP expression in A-KINACT and A-KINACT (T/A) dual stable cells. Scale bars, 50 μm. **c**, Histogram of EGFP intensity in A-KINACT dual stable cells by flow-cytometry.

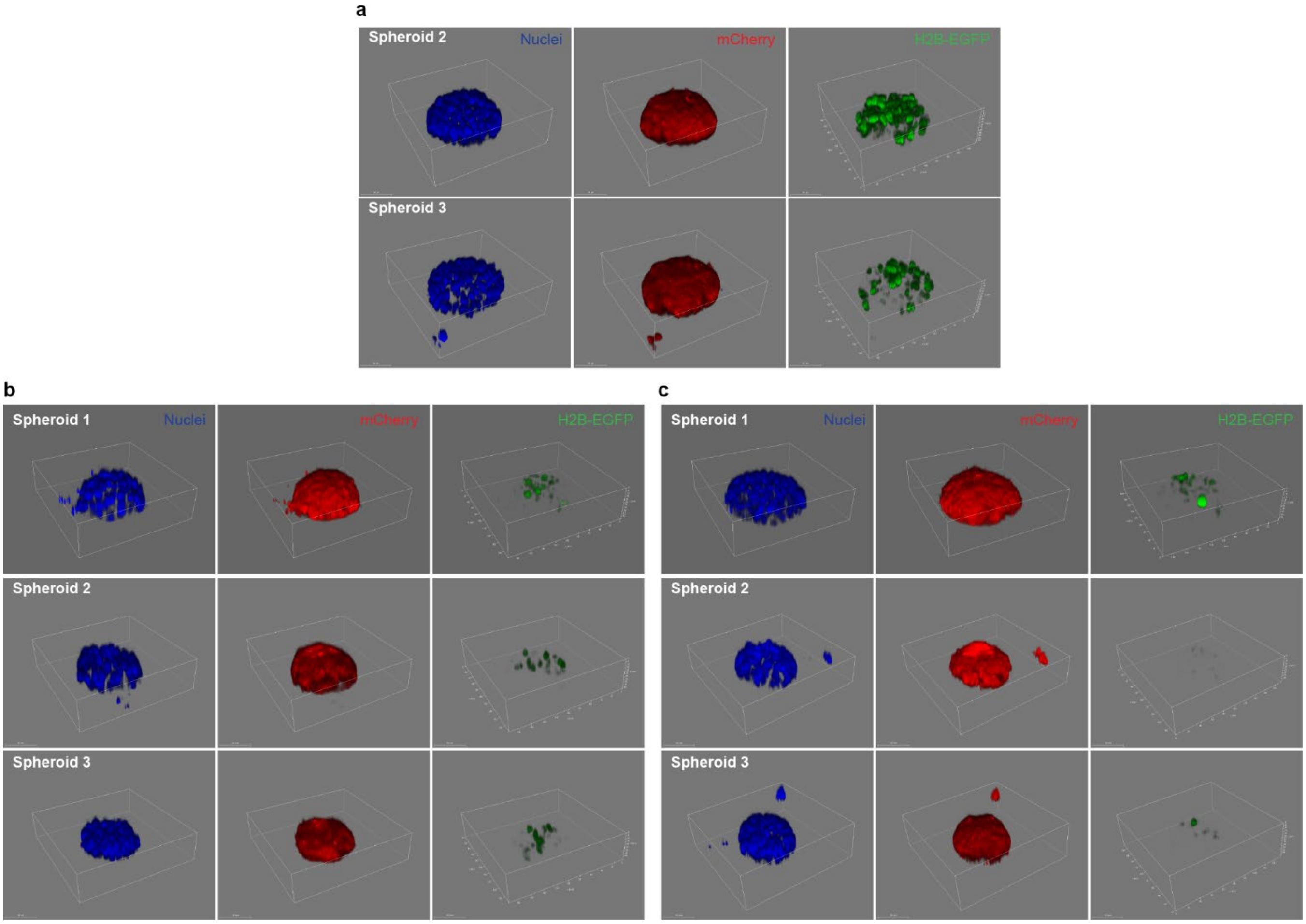

Supplementary Fig. 9 | Representative 3D imaging of basal PKA activity within HEK293T spheroids. **a**, Two replicates of 3D PKA activity imaging in A-KINACT spheroids. **b**, Three replicates of 3D PKA activity imaging in A-KINACT spheroids pretreated with H89. **c**, Three replicates of 3D PKA activity imaging in A-KINACT (T/A) spheroids.

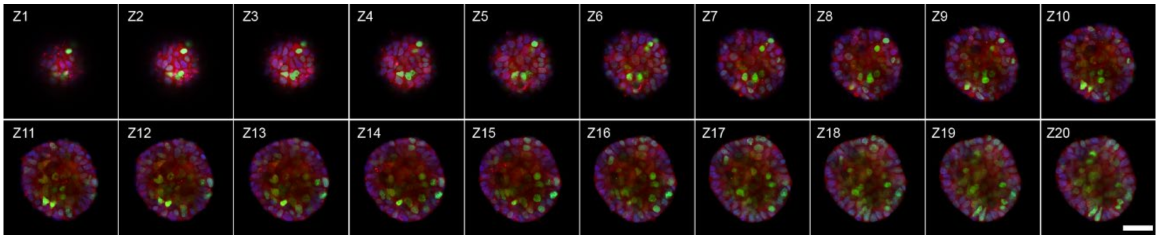

Supplementary Fig. 10 | Attenuation corrected images for triple-color confocal imaging of A-KINACT spheroid in the Z-direction (corresponding Fig. 3a). 20 layers of merged images of Hoechst-stained nuclei (blue), mCherry (red) and H2B-EGFP (green). Scale bar, 50 μm.

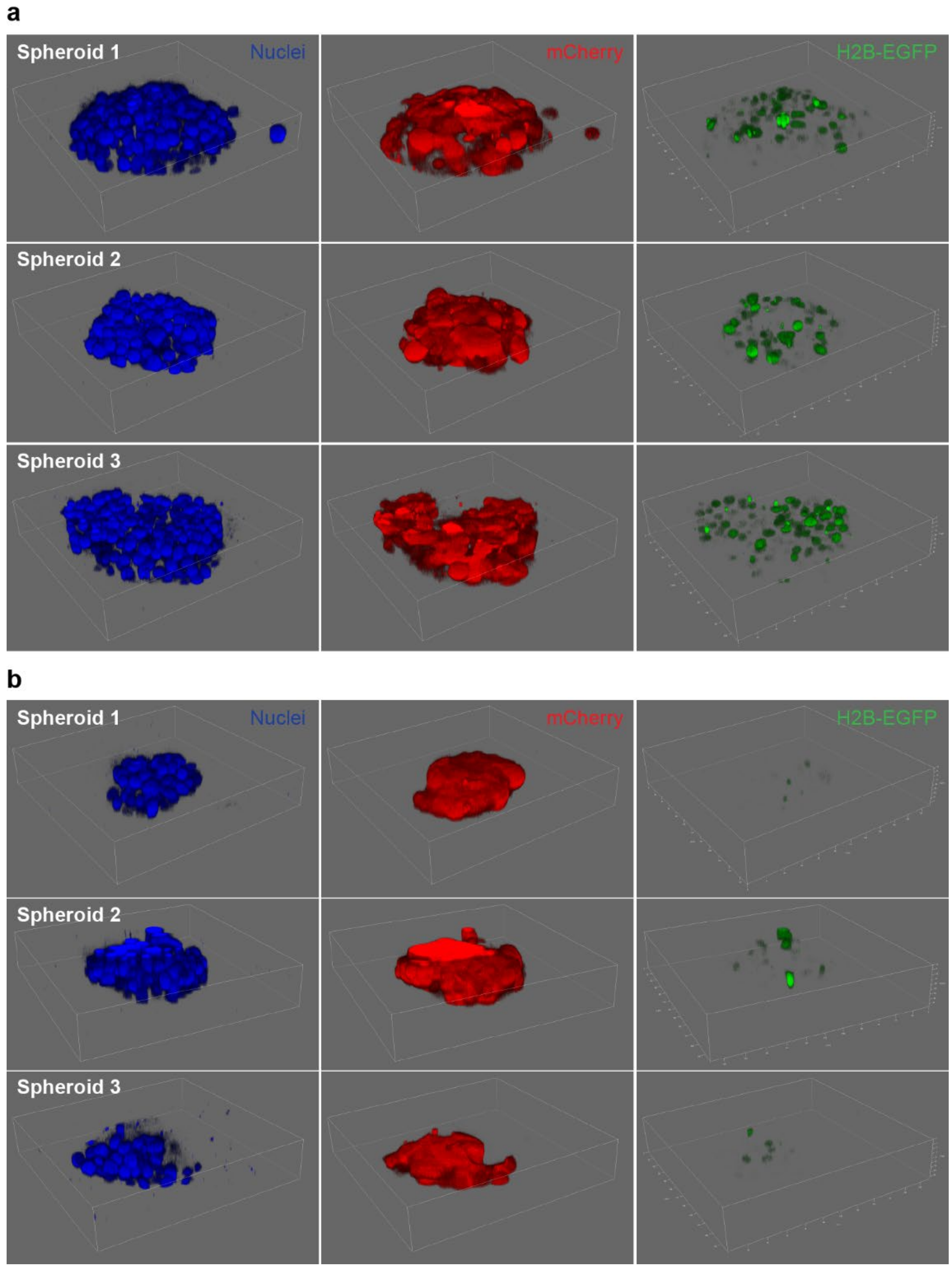

Supplementary Fig. 11 | Representative 3D imaging of basal PKA activity distribution within MCF-7 spheroids cultured in Matrigel. a, Three replicates of 3D PKA activity imaging in A-KINACT spheroids. **b**, Three replicates of 3D PKA activity imaging in A-KINACT (T/A) spheroids.

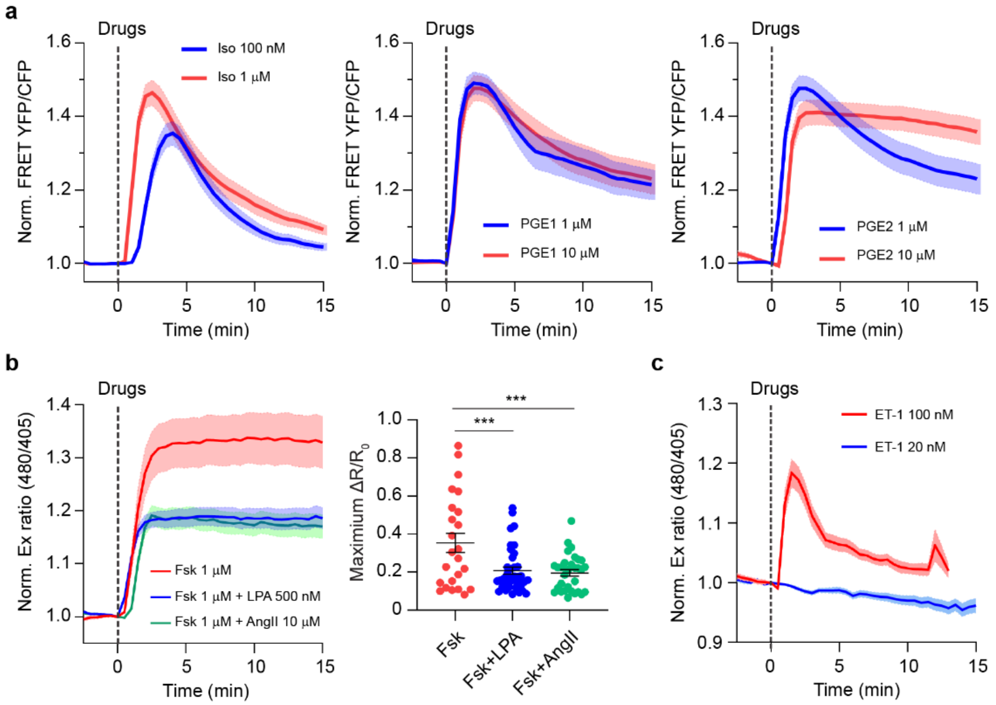

Supplementary Fig. 12 | Validation of PKA activity responding to Gα_s-, Gα_i- and Gα_q-linked drugs using AKARs. a, Representative average time courses of the PKA response stimulated with (left) 100 nM Iso (blue curve, n = 15 cells) and 1 µM Iso (red curve, n = 14 cells), (middle) 1 µM PGE1 (blue curve, n = 20 cells) and 10 µM PGE1 (red curve, n = 18 cells), (right) 1 µM PGE2 (blue curve, n = 18 cells) and 10 µM PGE2 (red curve, n = 20 cells) using AKAR4. 2 independent experiments were performed. **b**, Representative average time courses (left) and maximum responses ($\Delta R/R_0$, right) of the PKA activty stimulated with 1 µM Fsk only (red, n = 24 cells from 2 independent experiments), premixed 1 µM Fsk and 500 nM LPA (blue, ***P = 0.001, n = 38 cells from 3 independent experiments) and premixed 1 µM Fsk and 10 µM Ang II (green, ***P = 0.0007, n = 31 cells from 2 independent experiments) using ExRai-AKAR2. Statistical analysis was performed using ordinary one-way ANOVA followed by Dunnett's multiple-comparisons test. Data are mean ± s.e.m. **c**, Representative average time courses of the PKA response stimulated with 20 nM ET-1 (blue curve, n = 8 cells) and 100 nM ET-1 (red curve, n = 8 cells) using ExRai-AKAR2. 2 independent experiments were performed. For **a**, **b** and **c**, the solid lines represent the mean; shaded areas, s.e.m. Dashed lines indicate drug addition. Source data are provided as a Source Data file.

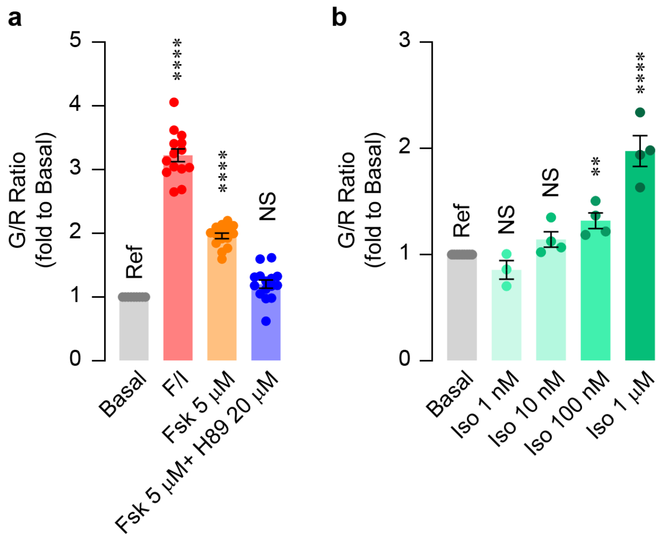

Supplementary Fig. 13 | Testing the sensitivity of the A-KINACT readout on a multi-well plate reader. **a**, A-KINACT induction of H2B-EGFP expression in response to no stimulation (Basal, n = 9 wells), and stimulation with F/I (50 μM/100 μM, positive control, n = 14 wells), low-dose Fsk (5 μM, n = 15 wells) and Fsk (5 μM) with H89 (20 μM) pretreatment (n = 15 wells). ****P < 0.0001. **b**, Iso dose response of A-KINACT. Basal (n = 9 wells), Iso (1 nM, n = 3 wells), Iso (10 nM, n = 4 wells), Iso (100 nM, **P = 0.008, n = 4 wells) and Iso (1 μM, ****P < 0.0001, n = 4 wells). Statistical analysis was performed using ordinary one-way ANOVA followed by Dunnett's multiple-comparisons test. NS, not significant. Data are mean ± s.e.m. Source data are provided as a Source Data file.

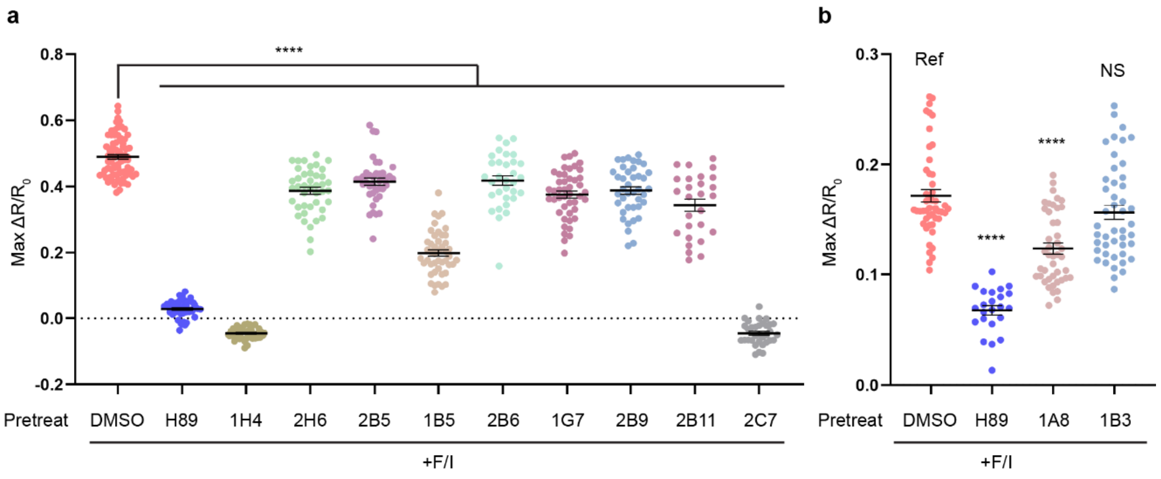

Supplementary Fig. 14 | Validation of inhibitory effects of 11 kinase inhibitors on PKA activity. a, Maximum responses ($\Delta R/R_0$) of GR-AKARev in HEK293T cells pretreated with each inhibitor (10 μM) followed by treatment with F/I (50 μM/100 μM). DMSO, n = 73 cells from 5 independent experiments. H89, n = 43 cells; 1H4, n = 39 cells; 2H6, n = 42 cells; 2B5, n = 39 cells; 1B5, n = 46 cells; 2B6, n = 32 cells; 1G7, n = 46 cells; 2B9, n = 40 cells; 2B11, n = 27 cells; 2C7, n = 33 cells; data from 3 independent experiments. **b**, Maximum responses ($\Delta R/R_0$) of Booster-PKA in HEK293T cells pretreated with each inhibitor (10 μM) followed by F/I (50 μM/100 μM). DMSO, n = 48 cells from 4 independent experiments; H89, n = 23 cells from 2 independent experiments; 1A8, n = 44 cells from 3 independent experiments; 1B3, n = 46 cells from 3 independent experiments. Statistical analysis was performed using ordinary one-way ANOVA followed by Dunnett's multiple-comparisons test. ****$P < 0.0001$. NS, not significant. Data are mean ± s.e.m. Source data are provided as a Source Data file.

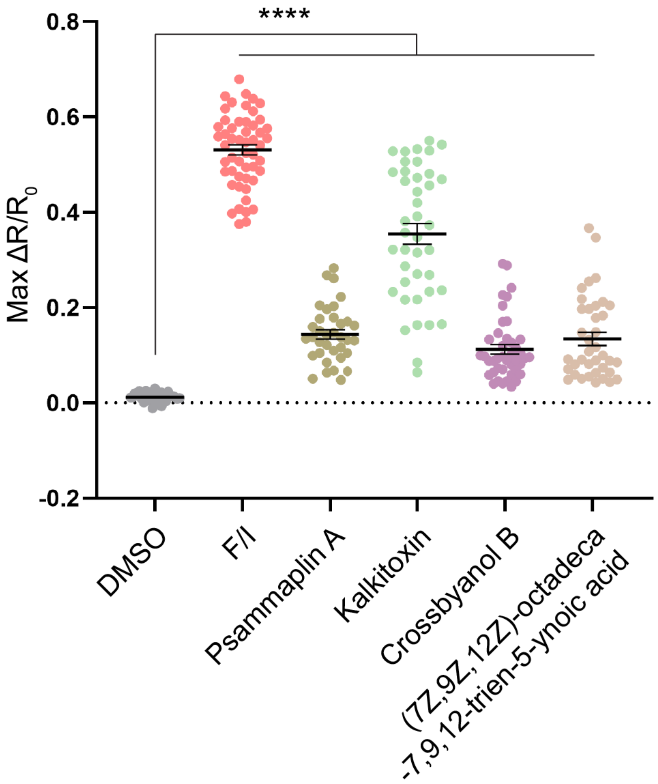

Supplementary Fig. 15 | Validation of 4 PKA activators from marine natural product library. Maximum responses ($\Delta R/R_0$) of AKAR3ev in HEK293T cells treated with each compound (2 µg ml^{-1}). F/I (50 µM/100 µM) treatment shown as a positive control. DMSO, n = 35 cells; F/I, n = 52 cells; Psa-A, n = 35 cells; Kal, n = 41 cells; Cro-B, n = 42 cells; 7Z, n = 39 cells. Statistical analysis was performed using ordinary one-way ANOVA followed by Dunnett's multiple-comparisons test. ****P < 0.0001. All data from 3 independent experiments. Data are mean ± s.e.m. Source data are provided as a Source Data file.

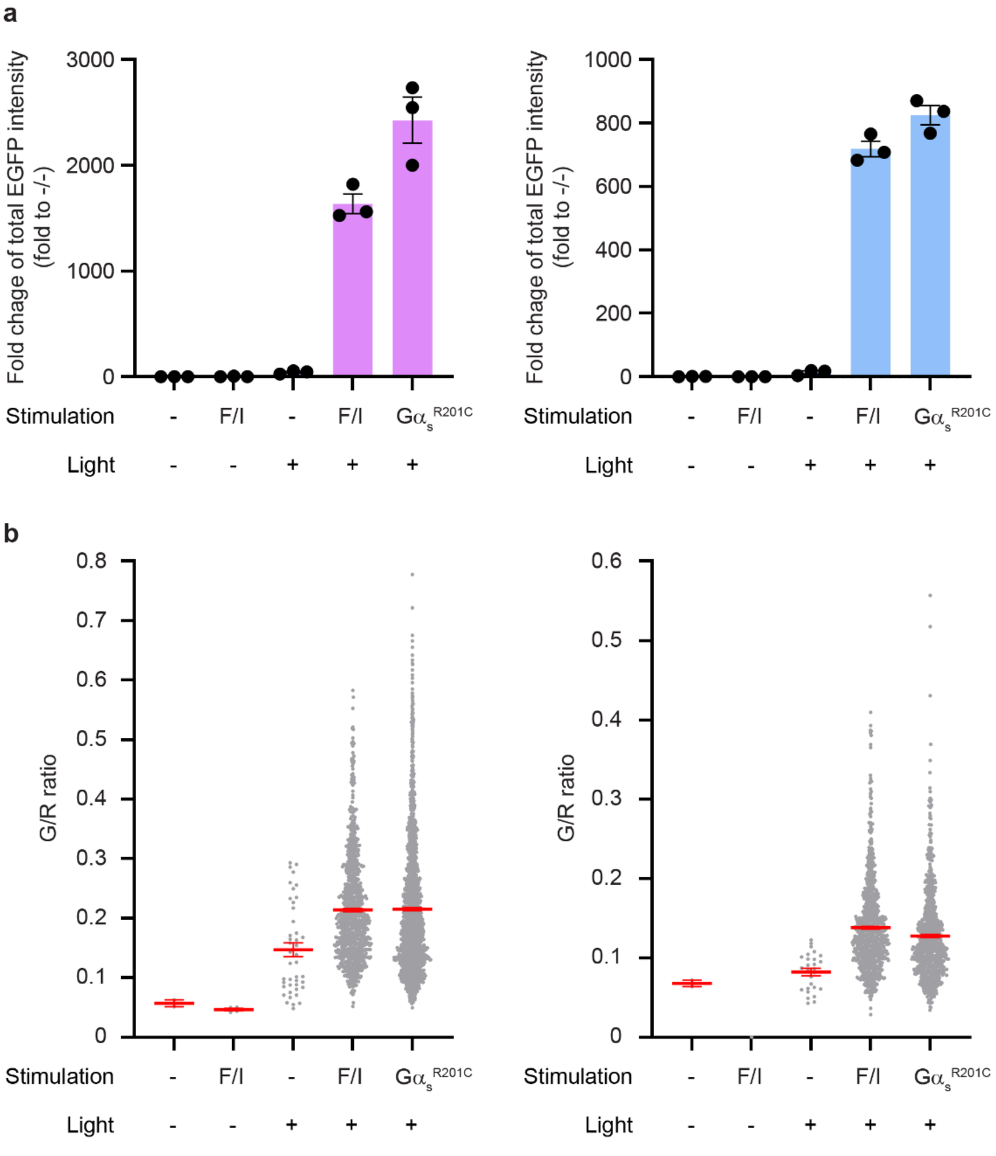

Supplementary Fig. 16 | Characterization of A-KINACT effector (PKI-EGFP) and A-KINACT control (EGFP) dual-stable cell lines. a, Statistical quantification of total EGFP intensity in A-KINACT effector cells (left) and A-KINACT control cells (right) under -F/I/-light, +F/I/-light, -F/I/+light, +F/I/+light and +$G\alpha_s^{R201C}$/+light conditions. n = 3 independent experiments. NS, not significant. Statistical analysis was performed using ordinary one-way ANOVA followed by Dunnett's multiple-comparisons test. **b**, G/R ratio of individual PKI-EGFP$^+$ cells (left, n = 2, n = 4, n = 43, n = 964 and n = 1913 cells from 3 independent experiments) and EGFP$^+$ cells (right, n = 2, n = 0, n = 27, n = 973 and n = 854 cells from 3 independent experiments). Data are mean ± s.e.m. Source data are provided as a Source Data file.

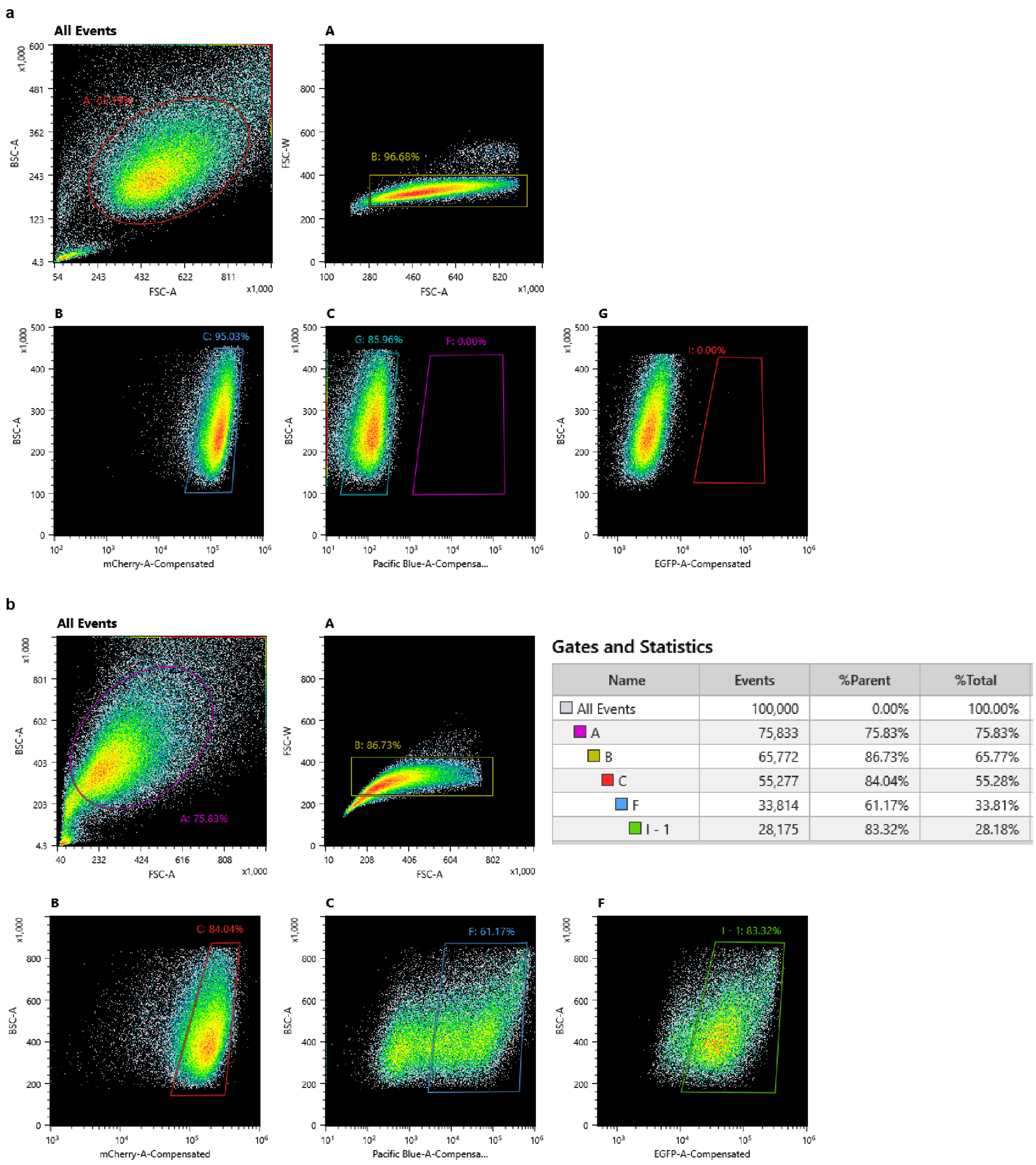

Gates and Statistics

Name	Events	%Parent	%Total
All Events	100,000	0.00%	100.00%
A	75,833	75.83%	75.83%
B	65,772	86.73%	65.77%
C	55,277	84.04%	55.28%
F	33,814	61.17%	33.81%
I - 1	28,175	83.32%	28.18%

Supplementary Fig. 17 | Procedure of triple-color FACS for sorting Gα$_s$^{R201C}-driven EGFP$^+$ cells. a, The distribution of triple-color fluorescence intensity of A-KINACT control cells without overexpressing mTagBFP2-Gα$_s$^{R201C} analyzed by flow cytometry. Gate A removing cell debris. Gate B indicating single cells. Gate C indicating mCherry positive cells. Gate G indicating mTagBFP2-Gα$_s$^{R201C} negative cells. Gate I indicating EGFP positive cells (0%). **b**, Gating strategy for sorting red/blue/green fluorescence positive cells. Gate A removing cell debris. Gate B indicating single cells. Gate C indicating mCherry positive cells. Gate F indicating mTagBFP2-Gα$_s$^{R201C} positive cells. Gate I-1 indicating EGFP positive cells which were finally collected.

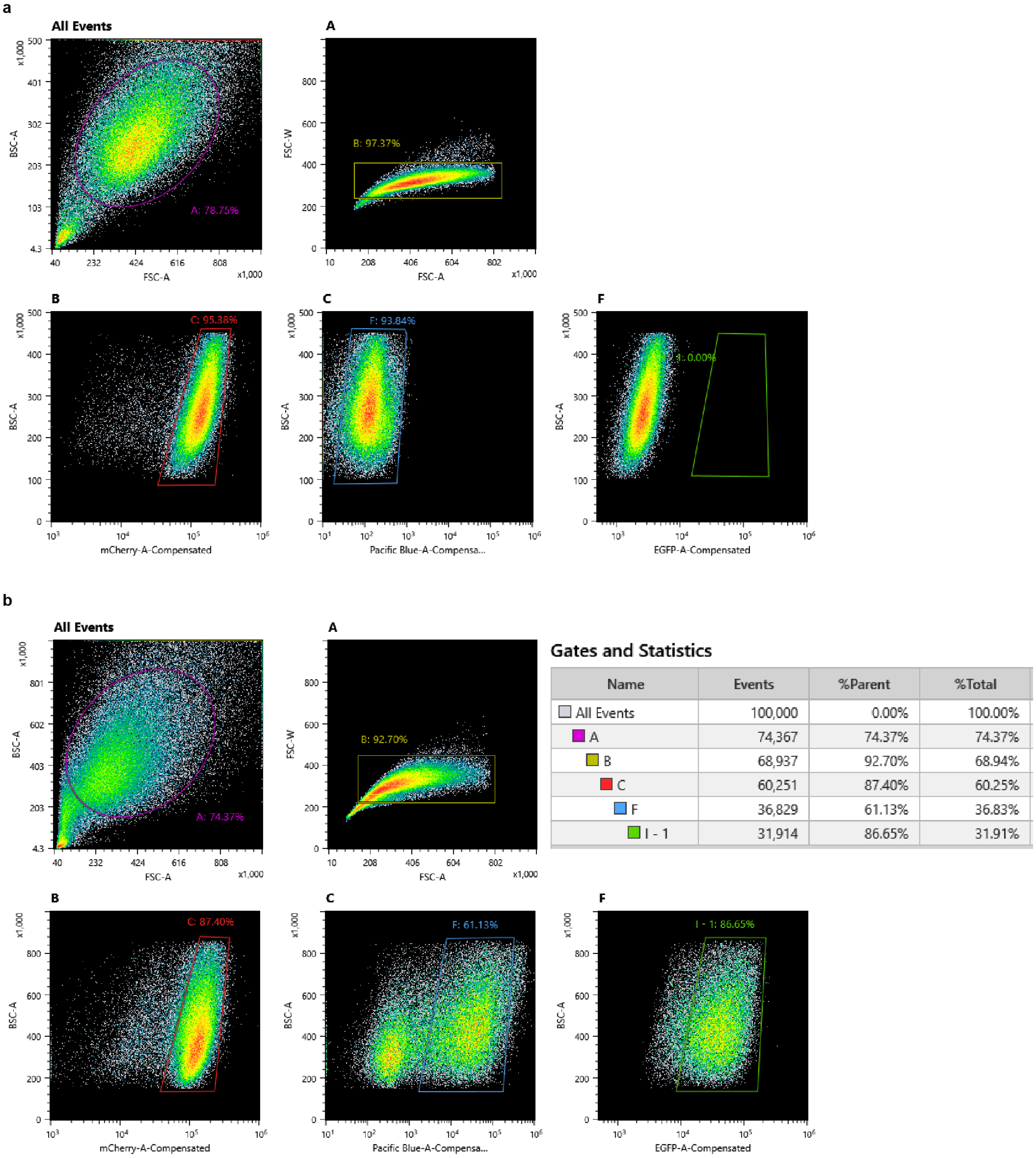

Name	Events	%Parent	%Total
All Events	100,000	0.00%	100.00%
A	74,367	74.37%	74.37%
B	68,937	92.70%	68.94%
C	60,251	87.40%	60.25%
F	36,829	61.13%	36.83%
I - 1	31,914	86.65%	31.91%

Supplementary Fig. 18 | Procedure of triple-color FACS for sorting Gα_s^{R201C}-driven PKI-EGFP$^+$ cells. **a**, The distribution of triple-color fluorescence intensity of A-KINACT effector cells without overexpressing mTagBFP2-Gα_s^{R201C} analyzed by flow cytometry. Gate A removing cell debris. Gate B indicating single cells. Gate C indicating mCherry positive cells. Gate F indicating mTagBFP2-Gα_s^{R201C} negative cells. Gate I indicating PKI-EGFP positive cells (0%). **b**, Gating strategy for sorting red/blue/green fluorescence positive cells. Gate A removing cell debris. Gate B indicating single cells. Gate C indicating mCherry positive cells. Gate F indicating mTagBFP2-Gα_s^{R201C} positive cells. Gate I-1 indicating PKI-EGFP positive cells which were finally collected.

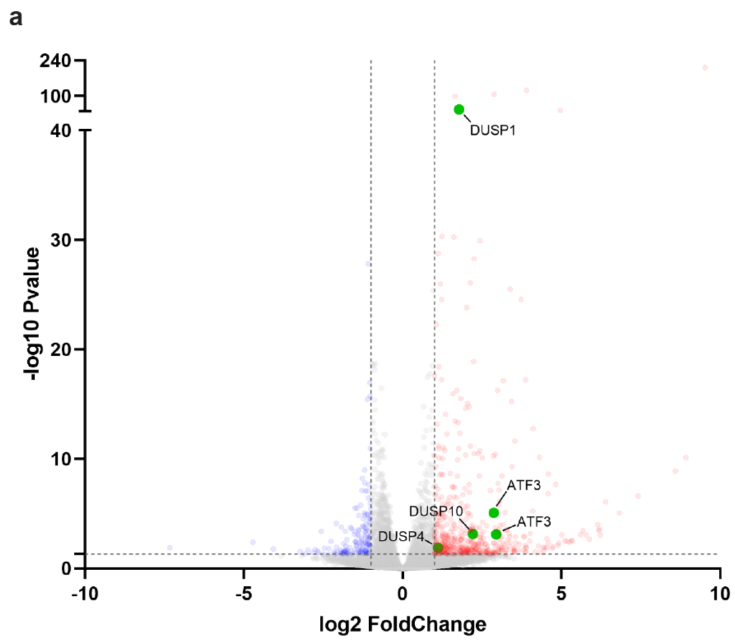

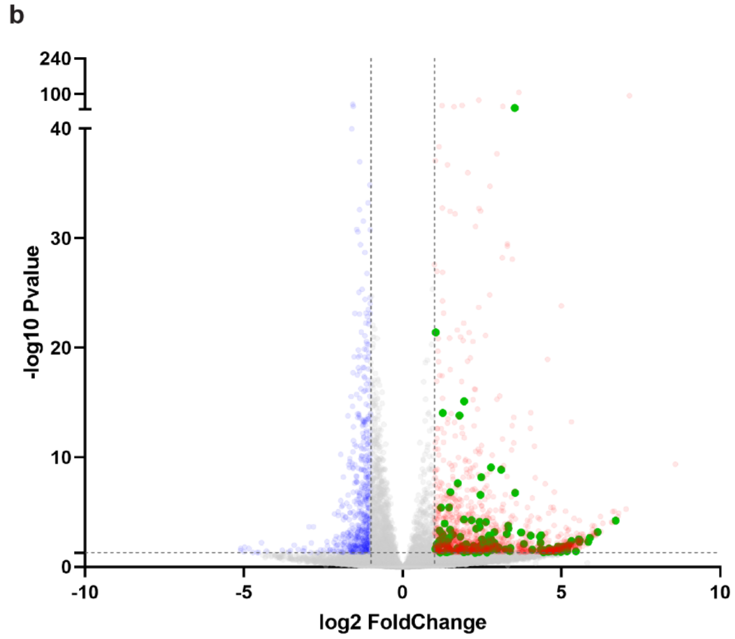

Supplementary Fig. 19 | Distribution of key PKA signatures and ERK signatures. a, Volcano plot showing relative enrichment of transcripts in A-KINACT control (EGFP) cells under $G\alpha_s^{R201C}$/light condition versus no $G\alpha_s^{R201C}$/no-light condition. Transcripts corresponding to 4 genes associated with "Negative regulation of ERK cascade" pathway are highlighted (green points). **b**, Volcano plot showing relative enrichment of transcripts in A-KINACT effector (PKI-EGFP) cells under $G\alpha_s^{R201C}$/light condition versus no $G\alpha_s^{R201C}$/no-light condition. Transcripts corresponding to 111 genes associated with "Cell cycle" pathway (sharing 60 genes associated with "Mitotic cell cycle proc." pathway; 68 genes associated with "Mitotic cell cycle" pathway; 87 genes associated with "Cell cycle proc." pathway) are highlighted (green points). Cut-off: P value < 0.05; Fold change > 2. See Supplementary Data 5 and 6 for gene lists.

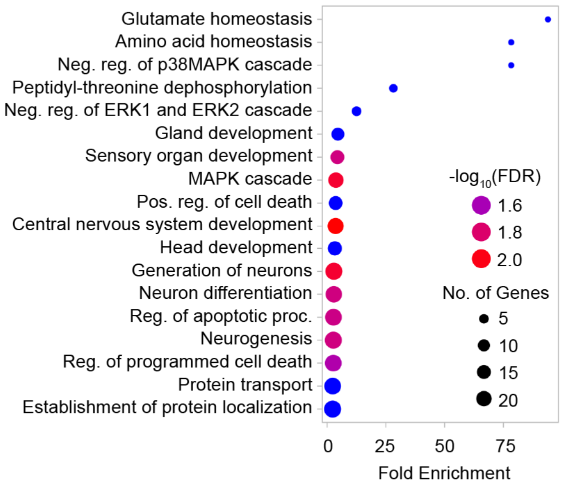

Supplementary Fig. 20 | GO analysis of 106 PKA-upregulated genes in A-KINACT control cells.

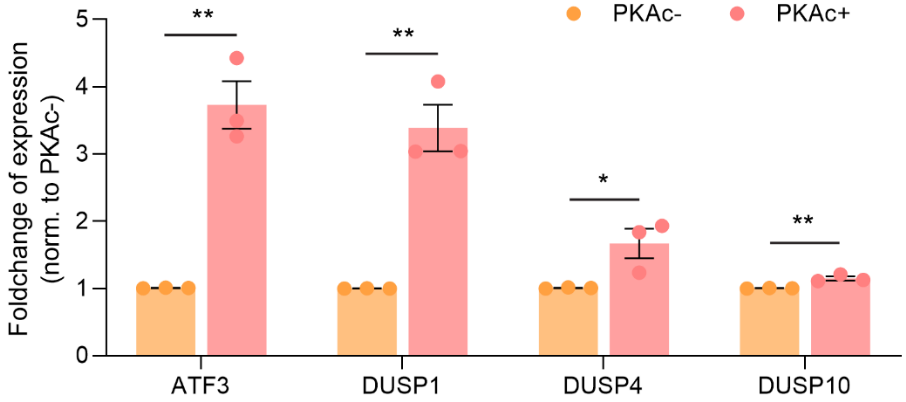

Supplementary Fig. 21 | Quantitative PCR (qPCR) analysis of identified PKA-upregulated genes under overexpression of PKA catalytic domain (PKAc). $**P$ = 0.0016 (ATF3), $**P$ = 0.0023 (DUSP1), $*P$ = 0.0388 (DUSP4), and $**P$ =0.0086 (DUSP10). Statistical analysis was performed using unpaired two-tailed Student *t*-test. Data from 3 independent experiments. Data are mean ± s.e.m. Source data are provided as a Source Data file.

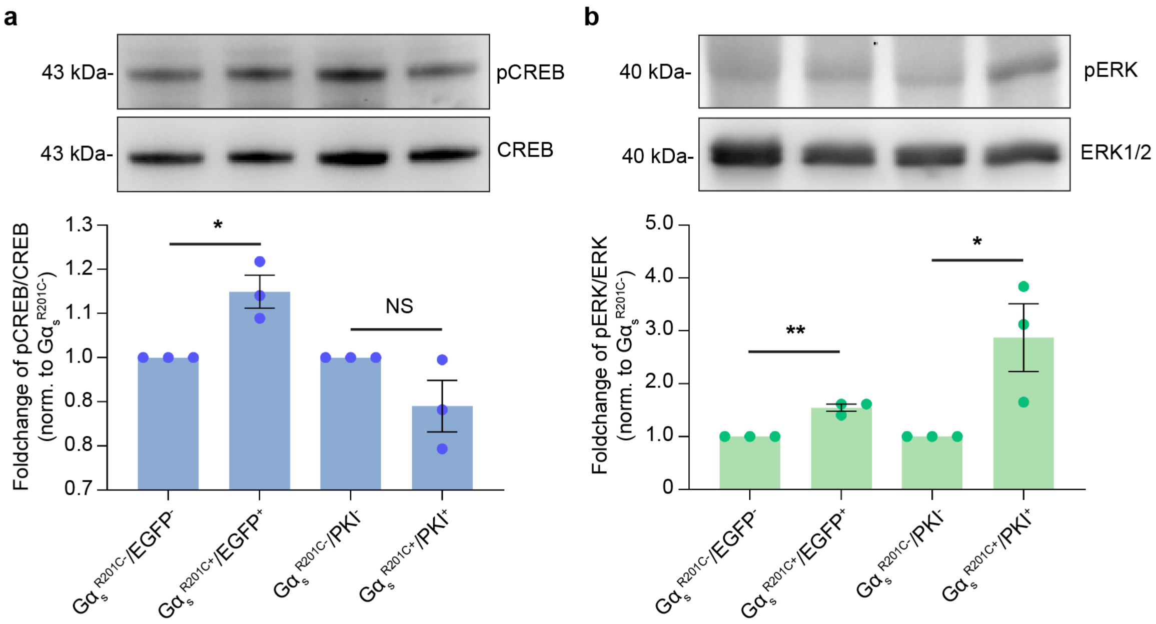

Supplementary Fig. 22 | Western blot characterization of A-KINACT effector manipulating $G\alpha_s^{R201C}$ signaling. a, Representative immunoblotting of CREB and pCREB in four conditions of cells. Bands at ~43 kDa correspond to CREB (top) and quantitative analysis of 3 independent experiments (bottom). *P = 0.0162 ($G\alpha_s^{R201C+}$/EGFP$^+$ vs. $G\alpha_s^{R201C-}$/EGFP$^-$). **b,** Representative immunoblotting of ERK1/2 and pERK in four conditions of cells. Bands at ~40 kDa correspond to ERK1/2 (top) and quantitative analysis of 3 independent experiments (bottom). **P = 0.0014 ($G\alpha_s^{R201C+}$/EGFP$^+$ vs. $G\alpha_s^{R201C-}$/EGFP$^-$), *P = 0.0439 ($G\alpha_s^{R201C+}$/PKI$^+$ vs. $G\alpha_s^{R201C-}$/PKI$^-$). Statistical analysis was performed using unpaired two-tailed Student *t*-test. NS, not significant. Data are mean ± s.e.m. Uncropped scans of western blots and source data are provided as a Source Data file.

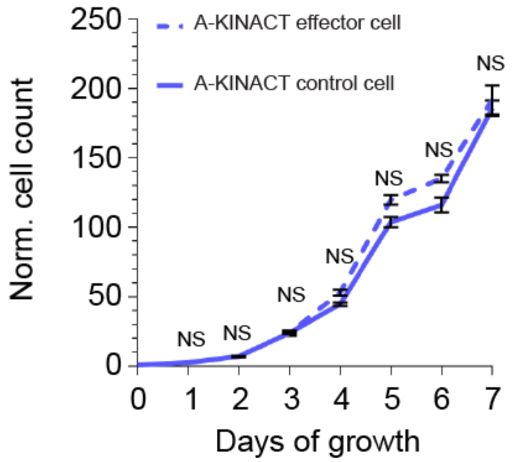

Supplementary Fig. 23 | Growth curves of A-KINACT effector cells and A-KINACT control cells without Gα_s^{R201C} overexpression and blue light illumination. There was no significant difference at each time point. n = 3 independent experiments. Statistical analysis was performed using unpaired two-tailed *t*-tests. NS, not significant. Data are mean ± s.e.m. Source data are provided as a Source Data file.

Sequences of constructs

A-KINACT Construct 1

METDTLLLWVLLLWVPGSTGDEQKLISEEDLNAVGQDTQEVIVVPHSLPFKVVVIS AILALVVLTIISLIILIMLWQKKPRGGSGGLEGM**LRRATLVD**ACGGGGSGGGGSGGGG RSGGSM**LQLPPLERLTLEMGESLFKGPRDYNPISSTICHLTNESDGHTTSLYGIGFGP FIITNKHLFRRNNGTLLVQSLHGVFKVKNTTTLQQHLIDGRDMIIIRMPKDFPPFPQ KLKFREPQREERICLVTTNFQ**ELAEKLAGLDINGGASG**SRATTLERIEKSFVITDPRLP DNPIIFVSDSFLQLTEYSREEILGRNCRFLQGPETDRATVRKIRDAIDNQTEVTVQLIN YTKSGKKFWNVFHLQPMRDYKGDVQYFIGVQLDGTERLHGAAEREAVCLVKKTA FQIAENLYFQGSRLDKSKVINSALELLNEVGIEGLTTRKLAQKLGVEQPTLYWHVK NKRALLDALAIEMLDRHHTHFCPLEGESWQDFLRNNAKSFRCALLSHRDGAKVHL GTRPTEKQYETLENQLAFLCQQGFSLENALYALSAVGHFTLGCVLEDQEHQVAKE ERETPTTDSMPPLLRQAIELFDHQGAEPAFLFGLELIICGLEKQLKCESGSAYSRART KNNYGSTIEGLLDLPDDDAPEEAGLAAPRLSFLPAGHTRRLSTAPPTDVSLGDELHL DGEDVAMAHADALDDFDLDMLGDGDSPGPGFTPHDSAPYGALDMADFEFEQMFT DALGIDEYGG***

Ig Kappa secretory element: 1-21 a.a.

Myc-epitope: 22-31 a.a.

PDGFRβ transmembrane helix: 32-81 a.a.

PKAsub: 91-98 a.a.

nuclear export signal (NES): 121-132 a.a.

TEVp-N: 133-250 a.a.

hLOV1: 268-405 a.a.

TEVseq-G: 406-412 a.a.

tTA-VP16: 413-746 a.a.

A-KINACT Construct 2

METDTLLLWVLLLWVPGSTGDEQKLISEEDLNAVGQDTQEVIVVPHSLPFKVVVIS
AILALVVLTIISLIILIMLWQKKPRGGSGGLEGM**LRRATLVD**ACGGGGSGGGGSGGGG
RSGGSM**LQLPPLERLTLEMGESLFKGPRDYNPISSTICHLTNESDGHTTSLYGIGFGP**
FIITNKHLFRRNNGTLLVQSLHGVFKVKNTTTLQQHLIDGRDMIIIRMPKDFPPFPQ
KLKFREPQREERICLVTTNFQELAEKLAGLDINGGASG**SRATTLERIEKSFVITDPRLP**
DNPIIFVSDSFLQLTEYSREEILGRNCRFLQGPETDRATVRKIRDAIDNQTEVTVQLIN
YTKSGKKFWNVFHLQPMRDYKGDVQYFIGVQLDGTERLHGAAEREAVCLVKKTA
FQIAENLYFQP**SRLDKSKVINSALELLNEVGIEGLTTRKLAQKLGVEQPTLYWHVK**
NKRALLDALAIEMLDRHHTHFCPLEGESWQDFLRNNAKSFRCALLSHRDGAKVHL
GTRPTEKQYETLENQLAFLCQQGFSLENALYALSAVGHFTLGCVLEDQEHQVAKE
ERETPTTDSMPPLLRQAIELFDHQGAEPAFLFGLELIICGLEKQLKCESGSAYSRART
KNNYGSTIEGLLDLPDDDAPEEAGLAAPRLSFLPAGHTRRLSTAPPTDVSLGDELHL
DGEDVAMAHADALDDFDLDMLGDGDSPGPGFTPHDSAPYGALDMADFEFEQMFT
DALGIDEYGG*

Ig Kappa secretory element: 1-21 a.a.

Myc-epitope: 22-31 a.a.

PDGFRβ transmembrane helix: 32-81 a.a.

PKAsub: 91-98 a.a.

nuclear export signal (NES): 121-132 a.a.

TEVp-N: 133-250 a.a.

hLOV1: 268-405 a.a.

TEVseq-P: 406-412 a.a.

tTA-VP16: 413-746 a.a.

A-KINACT Construct 3

METDTLLLWVLLLWVPGSTGDEQKLISEEDLNAVGQDTQEVIVVPHSLPFKVVVIS
AILALVVLTIISLIILIMLWQKKPRGGSGGLEGM**LRRATLVD**ACGGGGSGGGGSGGGG
RSGGSM**LQLPPLERLTLEMGESLFKGPRDYNPISSTICHLTNESDGHTTSLYGIGFGP**
FIITNKHLFRRNNGTLLVQSLHGVFKVKNTTTLQQHLIDGRDMIIIRMPKDFPPFPQ
KLKFREPQREERICLVTTNFQELAEKLAGLDINGGASG**SRATTLERIEKSFVITDPRLP**
DNPIIFVSDSFLQLTEYSREEILGRNCRFLQGPETDRATVRKIRDAIDNQTEVTVQLIN
YTKSGKKFWNVFHLQPMRDYKGDVQYFIGVQLDGTERLHGAAEREAVCLVKKTA
FQIAENLYFQM**SRLDKSKVINSALELLNEVGIEGLTTRKLAQKLGVEQPTLYWHVK**
NKRALLDALAIEMLDRHHTHFCPLEGESWQDFLRNNAKSFRCALLSHRDGAKVHL
GTRPTEKQYETLENQLAFLCQQGFSLENALYALSAVGHFTLGCVLEDQEHQVAKE
ERETPTTDSMPPLLRQAIELFDHQGAEPAFLFGLELIICGLEKQLKCESGSAYSRART
KNNYGSTIEGLLDLPDDDAPEEAGLAAPRLSFLPAGHTRRLSTAPPTDVSLGDELHL
DGEDVAMAHADALDDFDLDMLGDGDSPGPGFTPHDSAPYGALDMADFEFEQMFT
DALGIDEYGG*

Ig Kappa secretory element: 1-21 a.a.

Myc-epitope: 22-31 a.a.

PDGFRβ transmembrane helix: 32-81 a.a.

PKAsub: 91-98 a.a.

nuclear export signal (NES): 121-132 a.a.

TEVp-N: 133-250 a.a.

hLOV1: 268-405 a.a.

TEVseq-M: 406-412 a.a.

tTA-VP16: 413-746 a.a.

A-KINACT Construct 4

METDTLLLWVLLLWVPGSTGDEQKLISEEDLNAVGQDTQEVIVVPHSLPFKVVVIS
AILALVVLTIISLIILIMLWQKKPRGGSGGLEGM**LRRATLVD**ACGGGGSGGGGSGGGG
RSGGSM**LQLPPLERLTLEMGESLFKGPRDYNPISSTICHLTNESDGHTTSLYGIGFGP**
FIITNKHLFRRNNGTLLVQSLHGVFKVKNTTTLQQHLIDGRDMIIIRMPKDFPPFPQ
KLKFREPQREERICLVTTNFQELGSGSG**EFLATTLERIEKNFVITDPRLPDNPIIFASDS**
FLQLTEYSREEILGRNCRFLQGPETDRATVRKIRDAIDNQTEVTVQLINYTKSGKKF
WNVFHLQPMRDYKGDVQYFIGVQLDGTERLHGAAEREAVCLIKKTAFQIAENLYF
QGSRLDKSKVINSALELLNEVGIEGLTTRKLAQKLGVEQPTLYWHVKNKRALLDAL
AIEMLDRHHTHFCPLEGESWQDFLRNNAKSFRCALLSHRDGAKVHLGTRPTEKQY
ETLENQLAFLCQQGFSLENALYALSAVGHFTLGCVLEDQEHQVAKEERETPTTDSM
PPLLRQAIELFDHQGAEPAFLFGLELIICGLEKQLKCESGSAYSRARTKNNYGSTIEG
LLDLPDDDAPEEAGLAAPRLSFLPAGHTRRLSTAPPTDVSLGDELHLDGEDVAMAH
ADALDDFDLDMLGDGDSPGPGFTPHDSAPYGALDMADFEFEQMFTDALGIDEYGG*

Ig Kappa secretory element: 1-21 a.a.

Myc-epitope: 22-31 a.a.

PDGFRβ transmembrane helix: 32-81 a.a.

PKAsub: 91-98 a.a.

nuclear export signal (NES): 121-132 a.a.

TEVp-N: 133-250 a.a.

iLID: 258-396 a.a.

TEVseq-G: 397-403 a.a.

tTA-VP16: 404-737 a.a.

A-KINACT Construct 5

METDTLLLWVLLLWVPGSTGDEQKLISEEDLNAVGQDTQEVIVVPHSLPFKVVVIS
AILALVVLTIISLIILIMLWQKKPRGGSGGLEGMLRRATLVDACGGGGSGGGGSGGGG
RSGGSMLQLPPLERLTLEMGESLFKGPRDYNPISSTICHLTNESDGHTTSLYGIGFGP
FIITNKHLFRRNNGTLLVQSLHGVFKVKNTTTLQQHLIDGRDMIIIRMPKDFPPFPQ
KLKFREPQREERICLVTTNFQELAEKLAGLDINGGASGSRATTLERIEKSFVITDPRLP
DNPIIFVSDSFLQLTEYSREEILGRNCRFLQGPETDRATVRKIRDAIDNQTEVTVQLIN
YTKSGKKFWNLFHLQPMRDQKGDVQYFIGVQLDGTERVRDAAEREAVMLVKKTA
EEIDEAAKENLYFQMGGGSDYKDDDDKSRLDKSKVINSALELLNEVGIEGLTTRKL
AQKLGVEQPTLYWHVKNKRALLDALAIEMLDRHHTHFCPLEGESWQDFLRNNAK
SFRCALLSHRDGAKVHLGTRPTEKQYETLENQLAFLCQQGFSLENALYALSAVGHF
TLGCVLEDQEHQVAKEERETPTTDSMPPLLRQAIELFDHQGAEPAFLFGLELIICGL
EKQLKCESGSAYSRARTKNNYGSTIEGLLDLPDDDAPEEAGLAAPRLSFLPAGHTRR
LSTAPPTDVSLGDELHLDGEDVAMAHADALDDFDLDMLGDGDSPGPGFTPHDSAPY
GALDMADFEFEQMFTDALGIDEYGG*

Ig Kappa secretory element: 1-21 a.a.

Myc-epitope: 22-31 a.a.

PDGFRβ transmembrane helix: 32-81 a.a.

PKAsub: 91-98 a.a.

nuclear export signal (NES): 121-132 a.a.

TEVp-N: 133-250 a.a.

hLOV1: 268-409 a.a.

TEVseq-M: 410-416 a.a.

Flag-tTA-VP16: 421-762 a.a.

A-KINACT Construct 6

METDTLLLWVLLLWVPGSTGDEQKLISEEDLNAVGQDTQEVIVVPHSLPFKVVVIS
AILALVVLTIISLIILIMLWQKKPRGGSGGLEGM**LRRATLVD**ACGGGGSGGGGSGGGG
RSGGSM**LQLPPLERLTLE**ELAEKLAGLDINGGASG**SRATTLERIEKSFVITDPRLPDNP**
IIFVSDSFLQLTEYSREEILGRNCRFLQGPETDRATVRKIRDAIDNQTEVTVQLINYTK
SGKKFWNVFHLQPMRDYKGDVQYFIGVQLDGTERLHGAAEREAVCLVKKTAFQIA
ENLYFQGSRLDKSKVINSALELLNEVGIEGLTTRKLAQKLGVEQPTLYWHVKNKRA
LLDALAIEMLDRHHTHFCPLEGESWQDFLRNNAKSFRCALLSHRDGAKVHLGTRP
TEKQYETLENQLAFLCQQGFSLENALYALSAVGHFTLGCVLEDQEHQVAKEERETP
TTDSMPPLLRQAIELFDHQGAEPAFLFGLELIICGLEKQLKCESGSAYSRARTKNNY
GSTIEGLLDLPDDDAPEEAGLAAPRLSFLPAGHTRRLSTAPPTDVSLGDELHLDGED
VAMAHADALDDFDLDMLGDGDSPGPGFTPHDSAPYGALDMADFEFEQMFTDALGI
DEYGG*

Ig Kappa secretory element: 1-21 a.a.

Myc-epitope: 22-31 a.a.

PDGFRβ transmembrane helix: 32-81 a.a.

PKAsub: 91-98 a.a.

nuclear export signal (NES): 121-132 a.a.

hLOV1: 150-287 a.a.

TEVseq-G: 288-294 a.a.

tTA-VP16: 295-628 a.a.

A-KINACT Construct 7

M**EQKLISEEDLLQLPPLERLTLE**AT**MKFSQEQIGENIVCRVICTTGQIPIRDLSADISQ VLKEKRSIKKVWTFGRNPACDYHLGNISRLSNKHFQILLGEDGNLLLNDISTNGTW LNGQKVEKNSNQLLSQGDEITVGVGVESDILSLVIFINDKFKQCLEQNKVDR**LQELG GGGRSGGGGS**TKSMSSMVSDTSCTFPSSDGIFWKHWIQTKDGQCGSPLVSTRDGFIV GIHSASNFTNTNNYFTSVPKNFMELLTNQEAQQWVSGWRLNADSVLWGGHKVFM V***

Myc-epitope: 2-11 a.a.

nuclear export signal (NES): 12-23 a.a.

FHA1: 26-167 a.a.

TEVp-C: 183-284 a.a.

A-KINACT Construct 8

M**EQKLISEEDLLQLPPLERLTLE**AT**MKFSQEQIGENIVCRVICTTGQIPIRDLSADISQ VLKEKRSIKKVWTFGRNPACDYHLGNISRLSNKHFQILLGEDGNLLLNDISTNGTW LNGQKVEKNSNQLLSQGDEITVGVGVESDILSLVIFINDKFKQCLEQNKVDR**LQELG GGGRSGGGGS**MGESLFKGPRDYNPISSTICHLTNESDGHTTSLYGIGFGPFIITNKHLF RRNNGTLLVQSLHGVFKVKNTTTLQQHLIDGRDMIIIRMPKDFPPFPQKLKFREPQ REERICLVTTNFQTKSMSSMVSDTSCTFPSSDGIFWKHWIQTKDGQCGSPLVSTRDG FIVGIHSASNFTNTNNYFTSVPKNFMELLTNQEAQQWVSGWRLNADSVLWGGHKV FMV***

Myc-epitope: 2-11 a.a.

nuclear export signal (NES): 12-23 a.a.

FHA1: 26-167 a.a.

TEVp: 183-402 a.a.

Mito-A-KINACT

MAIQLRSLFPLALPGMLALLGWWWFFSRKKEQKLISEEDLGGSGGLEGM**LRRATL**
VDACGGGGSGGGGSGGGGRSGGSM**LQLPPLERLTLEMGESLFKGPRDYNPISSTICH**
LTNESDGHTTSLYGIGFGPFIITNKHLFRRNNGTLLVQSLHGVFKVKNTTTLQQHLI
DGRDMIIIRMPKDFPPFPQKLKFREPQREERICLVTTNFQELAEKLAGLDINGGASG**S**
RATTLERIEKSFVITDPRLPDNPIIFVSDSFLQLTEYSREEILGRNCRFLQGPETDRAT
VRKIRDAIDNQTEVTVQLINYTKSGKKFWNVFHLQPMRDYKGDVQYFIGVQLDGT
ERLHGAAEREAVCLVKKTAFQIAENLYFQGSRLDKSKVINSALELLNEVGIEGLTTR
KLAQKLGVEQPTLYWHVKNKRALLDALAIEMLDRHHTHFCPLEGESWQDFLRNN
AKSFRCALLSHRDGAKVHLGTRPTEKQYETLENQLAFLCQQGFSLENALYALSAVG
HFTLGCVLEDQEHQVAKEERETPTTDSMPPLLRQAIELFDHQGAEPAFLFGLELIIC
GLEKQLKCESGSAYSRARTKNNYGSTIEGLLDLPDDDAPEEAGLAAPRLSFLPAGHT
RRLSTAPPTDVSLGDELHLDGEDVAMAHADALDDFDLDMLGDGDSPGPGFTPHDS
APYGALDMADFEFEQMFTDALGIDEYGG*

DAKAP1-tag: 1-30 a.a.

Myc-epitope: 31-40 a.a.

PKAsub: 50-57 a.a.

nuclear export signal (NES): 80-91 a.a.

TEVp-N: 92-209 a.a.

hLOV1: 227-364 a.a.

TEVseq-G: 365-371 a.a.

tTA-VP16: 372-705 a.a.

C-KINACT Component 1

METDTLLLWVLLLWVPGSTGDEQKLISEEDLNAVGQDTQEVIVVPHSLPFKVVVIS AILALVVLTIISLIILIMLWQKKPRGGSGGLEGM**RFRRFQTLKDKAKA**ACGGGGSGG GGSGGGGRSGGSM**LQLPPLERLTLEMGESLFKGPRDYNPISSTICHLTNESDGHTTSL YGIGFGPFIITNKHLFRRNNGTLLVQSLHGVFKVKNTTTLQQHLIDGRDMIIIRMPK DFPPFPQKLKFREPQREERICLVTTNFQ**ELAEKLAGLDINGGASG**SRATTLERIEKSFV ITDPRLPDNPIIFVSDSFLQLTEYSREEILGRNCRFLQGPETDRATVRKIRDAIDNQTE VTVQLINYTKSGKKFWNVFHLQPMRDYKGDVQYFIGVQLDGTERLHGAAEREAVC LVKKTAFQIA**ENLYFQG**SRLDKSKVINSALELLNEVGIEGLTTRKLAQKLGVEQPTL YWHVKNKRALLDALAIEMLDRHHTHFCPLEGESWQDFLRNNAKSFRCALLSHRDG AKVHLGTRPTEKQYETLENQLAFLCQQGFSLENALYALSAVGHFTLGCVLEDQEH QVAKEERETPTTDSMPPLLRQAIELFDHQGAEPAFLFGLELIICGLEKQLKCESGSA YSRARTKNNYGSTIEGLLDLPDDDAPEEAGLAAPRLSFLPAGHTRRLSTAPPTDVSL GDELHLDGEDVAMAHADALDDFDLDMLGDGDSPGPGFTPHDSAPYGALDMADFEF EQMFTDALGIDEYGG***

Ig Kappa secretory element: 1-21 a.a.

Myc-epitope: 22-31 a.a.

PDGFRβ transmembrane helix: 32-81 a.a.

PKCsub: 91-104 a.a.

nuclear export signal (NES): 127-138 a.a.

TEVp-N: 139-256 a.a.

hLOV1: 274-411 a.a.

TEVseq-G: 412-418 a.a.

tTA-VP16: 419-752 a.a.

C-KINACT Component 2

Same with A-KINACT construct 6

F-KINACT Component 1

METDTLLLWVLLLWVPGSTGDEQKLISEEDLNAVGQDTQEVIVVPHSLPFKVVVIS
AILALVVLTIISLIILIMLWQKKPRGGSGGLEGMEKIEGTYGVVACGGGGSGGGGSGG
GGRSGGSMLQLPPLERLTLEMGESLFKGPRDYNPISSTICHLTNESDGHTTSLYGIGF
GPFIITNKHLFRRNNGTLLVQSLHGVFKVKNTTTLQQHLIDGRDMIIIRMPKDFPPFP
QKLKFREPQREERICLVTTNFQELAEKLAGLDINGGASGSRATTLERIEKSFVITDPRL
PDNPIIFVSDSFLQLTEYSREEILGRNCRFLQGPETDRATVRKIRDAIDNQTEVTVQLI
NYTKSGKKFWNVFHLQPMRDYKGDVQYFIGVQLDGTERLHGAAEREAVCLVKKT
AFQIAENLYFQGSRLDKSKVINSALELLNEVGIEGLTTRKLAQKLGVEQPTLYWHV
KNKRALLDALAIEMLDRHHTHFCPLEGESWQDFLRNNAKSFRCALLSHRDGAKVH
LGTRPTEKQYETLENQLAFLCQQGFSLENALYALSAVGHFTLGCVLEDQEHQVAK
EERETPTTDSMPPLLRQAIELFDHQGAEPAFLFGLELIICGLEKQLKCESGSAYSRAR
TKNNYGSTIEGLLDLPDDDAPEEAGLAAPRLSFLPAGHTRRLSTAPPTDVSLGDELH
LDGEDVAMAHADALDDFDLDMLGDGDSPGPGFTPHDSAPYGALDMADFEFEQMFT
DALGIDEYGG*

Ig Kappa secretory element: 1-21 a.a.

Myc-epitope: 22-31 a.a.

PDGFRβ transmembrane helix: 32-81 a.a.

Fynsub: 91-100 a.a.

nuclear export signal (NES): 123-134 a.a.

TEVp-N: 135-252 a.a.

hLOV1: 270-407 a.a.

TEVseq-G: 408-414 a.a.

tTA-VP16: 415-748 a.a.

F-KINACT Component 2

MEQKLISEEDLLQLPPLERLTLEATMWYFGKITRRESERLLLNPENPRGTFLVRESE
TTKGAYALSVSDFDNAKGLNVKHYKIRKLDSGGFYITSRTQFSSLQQLVAYYSKHA
DGLCHRLTNVLQELGGGGRSGGGGSTKSMSSMVSDTSCTFPSSDGIFWKHWIQTKD
GQCGSPLVSTRDGFIVGIHSASNFTNTNNYFTSVPKNFMELLTNQEAQQWVSGWRL
NADSVLWGGHKVFMV*

Myc-epitope: 2-11 a.a.

nuclear export signal (NES): 12-23 a.a.

SH2(C185A): 26-123 a.a.

TEVp-C: 139-240 a.a.

TetO-reporters

TetO-H2B-EGFP

cgagtttaccactccctatcagtgatagagaaaagtgaaagtcgagtttaccactccctatcagtgatagagaaaagtgaaagtcgagtttacc
actccctatcagtgatagagaaaagtgaaagtcgagtttaccactccctatcagtgatagagaaaagtgaaagtcgagtttaccactccctatc
agtgatagagaaaagtgaaagtcgagtttaccactccctatcagtgatagagaaaagtgaaagtcgagtttaccactccctatcagtgataga
gaaaagtgaaagtcgagtttaccactccctatcagtgatagagaaaagtgaaagtcgagtttaccactccctatcagtgatagagaaaagtga
aagtcgagtttaccactccctatcagtgatagagaaaagtgaaagtcgagtttaccactccctatcagtgatagagaaaagtgaaagtcgagtt
taccactccctatcagtgatagagaaaagtgaaagtcgagtttaccactccctatcagtgatagagaaaagtgaaagtcgagctcggtacgct
atggcatgcatgtgtcgcgacctgcaggccctgaagttcatctgcaccaccggcaagctgcccgtgccctggcccaccctcgtgaccaccc
tgacctggggcgtgcagtgcttcgcccgctaccccgaccacatgaagcagcacgacttcttcaagtccgccatgcccgaaggctacgtcca
ggagcgcaccatcttcttcaaggacgacggcaactacaagacccgcgccgaggtgaagttcgagggcgacaccctggtgaaccgcatcg
agctgaagggcatcgacttcaaggaggacggcaacatcctggggcacaagctggagtacaacgccatcagcgacaacgtctatatcaccg
ccgacaagcagaagaacggcatcaaggccaacttcaagagctagccctatataagcagagctcgtttagtgaaccgtcagatcgcctggag
acgccatccacgctgttttgacctccatagaagacaccgggaccgatccagcctccgcggccccggtaccgaattcaaggcctctcgagcc
tctagaaggtggcggaATGCCAGAGCCAGCGAAGTCTGCTCCCGCCCCGAAAAAGGGCTCCA
AGAAGGCGGTGACTAAGGCGCAGAAGAAAGGCGGCAAGAAGCGCAAGCGCAGCCG
CAAGGAGAGCTATTCCATCTATGTGTACAAGGTTCTGAAGCAGGTCCACCCTGACAC
CGGCATTTCGTCCAAGGCCATGGGCATCATGAATTCGTTTGTGAACGACATTTTCGA
GCGCATCGCAGGTGAGGCTTCCCGCCTGGCGCATTACAACAAGCGCTCGACCATCAC
CTCCAGGGAGATCCAGACGGCCGTGCGCCTGCTGCTGCCTGGGGAGTTGGCCAAGCA
CGCCGTGTCCGAGGGTACTAAGGCCATCACCAAGTACACCAGCGCTAAGgatccaccggtc
gccaccATGGTGAGCAAGGGCGAGGAGCTGTTCACCGGGGTGGTGCCCATCCTGGTCGA
GCTGGACGGCGACGTAAACGGCCACAAGTTCAGCGTGTCCGGCGAGGGCGAGGGCG
ATGCCACCTACGGCAAGCTGACCCTGAAGTTCATCTGCACCACCGGCAAGCTGCCCG
TGCCCTGGCCCACCCTCGTGACCACCCTGACCTACGGCGTGCAGTGCTTCAGCCGCT
ACCCCGACCACATGAAGCAGCACGACTTCTTCAAGTCCGCCATGCCCGAAGGCTACG
TCCAGGAGCGCACCATCTTCTTCAAGGACGACGGCAACTACAAGACCCGCGCCGAG
GTGAAGTTCGAGGGCGACACCCTGGTGAACCGCATCGAGCTGAAGGGCATCGACTTC
AAGGAGGACGGCAACATCCTGGGGCACAAGCTGGAGTACAACTACAACAGCCACAA
CGTCTATATCATGGCCGACAAGCAGAAGAACGGCATCAAGGTGAACTTCAAGATCC
GCCACAACATCGAGGACGGCAGCGTGCAGCTCGCCGACCACTACCAGCAGAACACC
CCCATCGGCGACGGCCCCGTGCTGCTGCCCGACAACCACTACCTGAGCACCCAGTCC
GCCCTGAGCAAAGACCCCAACGAGAAGCGCGATCACATGGTCCTGCTGGAGTTCGTG
ACCGCCGCCGGGATCACTCTCGGCATGGACGAGCTGTACAAGTAG

TetO-PKI-EGFP

cgagtttaccactccctatcagtgatagagaaaagtgaaagtcgagtttaccactccctatcagtgatagagaaaagtgaaagtcgagtttacc
actccctatcagtgatagagaaaagtgaaagtcgagtttaccactccctatcagtgatagagaaaagtgaaagtcgagtttaccactccctatc
agtgatagagaaaagtgaaagtcgagtttaccactccctatcagtgatagagaaaagtgaaagtcgagtttaccactccctatcagtgataga
gaaaagtgaaagtcgagtttaccactccctatcagtgatagagaaaagtgaaagtcgagtttaccactccctatcagtgatagagaaaagtga
aagtcgagtttaccactccctatcagtgatagagaaaagtgaaagtcgagtttaccactccctatcagtgatagagaaaagtgaaagtcgagtt
taccactccctatcagtgatagagaaaagtgaaagtcgagtttaccactccctatcagtgatagagaaaagtgaaagtcgagctcggtacgct
atggcatgcatgtgtcgcgacctgcaggccctgaagttcatctgcaccaccggcaagctgcccgtgccctggcccaccctcgtgaccaccc
tgacctggggcgtgcagtgcttcgcccgctaccccgaccacatgaagcagcacgacttcttcaagtccgccatgcccgaaggctacgtcca
ggagcgcaccatcttcttcaaggacgacggcaactacaagacccgcgccgaggtgaagttcgagggcgacaccctggtgaaccgcatcg
agctgaagggcatcgacttcaaggaggacggcaacatcctggggcacaagctggagtacaacgccatcagcgacaacgtctatatcaccg
ccgacaagcagaagaacggcatcaaggccaacttcaagagctagccctatataagcagagctcgtttagtgaaccgtcagatcgcctggag
acgccatccacgctgttttgacctccatagaagacaccgggaccgatccagcctccgcggccccggtaccgaattcaaggcctctcgagcc
tctagaaggtggcggaATGACATATGCAGATTTTATTGCTTCAGGAAGAACAGGTAGAAGAA
ATGCAATATGGgatccaccggtcgccaccATGGTGAGCAAGGGCGAGGAGCTGTTCACCGGGG
TGGTGCCCATCCTGGTCGAGCTGGACGGCGACGTAAACGGCCACAAGTTCAGCGTGT
CCGGCGAGGGCGAGGGCGATGCCACCTACGGCAAGCTGACCCTGAAGTTCATCTGC
ACCACCGGCAAGCTGCCCGTGCCCTGGCCCACCCTCGTGACCACCCTGACCTACGGC
GTGCAGTGCTTCAGCCGCTACCCCGACCACATGAAGCAGCACGACTTCTTCAAGTCC
GCCATGCCCGAAGGCTACGTCCAGGAGCGCACCATCTTCTTCAAGGACGACGGCAAC
TACAAGACCCGCGCCGAGGTGAAGTTCGAGGGCGACACCCTGGTGAACCGCATCGA
GCTGAAGGGCATCGACTTCAAGGAGGACGGCAACATCCTGGGGCACAAGCTGGAGT
ACAACTACAACAGCCACAACGTCTATATCATGGCCGACAAGCAGAAGAACGGCATC
AAGGTGAACTTCAAGATCCGCCACAACATCGAGGACGGCAGCGTGCAGCTCGCCGA
CCACTACCAGCAGAACACCCCCATCGGCGACGGCCCCGTGCTGCTGCCCGACAACCA
CTACCTGAGCACCCAGTCCGCCCTGAGCAAAGACCCCAACGAGAAGCGCGATCACA
TGGTCCTGCTGGAGTTCGTGACCGCCGCCGGGATCACTCTCGGCATGGACGAGCTGT
ACAAGTAG

TetO-EGFP

cgagtttaccactccctatcagtgatagagaaaagtgaaagtcgagtttaccactccctatcagtgatagagaaaagtgaaagtcgagtttacc
actccctatcagtgatagagaaaagtgaaagtcgagtttaccactccctatcagtgatagagaaaagtgaaagtcgagtttaccactccctatc
agtgatagagaaaagtgaaagtcgagtttaccactccctatcagtgatagagaaaagtgaaagtcgagtttaccactccctatcagtgataga
gaaaagtgaaagtcgagtttaccactccctatcagtgatagagaaaagtgaaagtcgagtttaccactccctatcagtgatagagaaaagtga
aagtcgagtttaccactccctatcagtgatagagaaaagtgaaagtcgagtttaccactccctatcagtgatagagaaaagtgaaagtcgagtt
taccactccctatcagtgatagagaaaagtgaaagtcgagtttaccactccctatcagtgatagagaaaagtgaaagtcgagctcggtacgct
atggcatgcatgtgtcgcgacctgcaggccctgaagttcatctgcaccaccggcaagctgcccgtgccctggcccaccctcgtgaccaccc
tgacctggggcgtgcagtgcttcgcccgctaccccgaccacatgaagcagcacgacttcttcaagtccgccatgcccgaaggctacgtcca
ggagcgcaccatcttcttcaaggacgacggcaactacaagacccgcgccgaggtgaagttcgagggcgacaccctggtgaaccgcatcg
agctgaagggcatcgacttcaaggaggacggcaacatcctggggcacaagctggagtacaacgccatcagcgacaacgtctatatcaccg
ccgacaagcagaagaacggcatcaaggccaacttcaagagctagccctatataagcagagctcgtttagtgaaccgtcagatcgcctggag
acgccatccacgctgttttgacctccatagaagacaccgggaccgatccagcctccgcggccccggtaccgaattcaaggcctctcgagcc
tctagaaggtggcggaATGGTGAGCAAGGGCGAGGAGCTGTTCACCGGGGTGGTGCCCATCC
TGGTCGAGCTGGACGGCGACGTAAACGGCCACAAGTTCAGCGTGTCCGGCGAGGGC
GAGGGCGATGCCACCTACGGCAAGCTGACCCTGAAGTTCATCTGCACCACCGGCAAG
CTGCCCGTGCCCTGGCCCACCCTCGTGACCACCCTGACCTACGGCGTGCAGTGCTTCA
GCCGCTACCCCGACCACATGAAGCAGCACGACTTCTTCAAGTCCGCCATGCCCGAAG
GCTACGTCCAGGAGCGCACCATCTTCTTCAAGGACGACGGCAACTACAAGACCCGCG
CCGAGGTGAAGTTCGAGGGCGACACCCTGGTGAACCGCATCGAGCTGAAGGGCATC
GACTTCAAGGAGGACGGCAACATCCTGGGGCACAAGCTGGAGTACAACTACAACAG
CCACAACGTCTATATCATGGCCGACAAGCAGAAGAACGGCATCAAGGTGAACTTCA
AGATCCGCCACAACATCGAGGACGGCAGCGTGCAGCTCGCCGACCACTACCAGCAG
AACACCCCCATCGGCGACGGCCCCGTGCTGCTGCCCGACAACCACTACCTGAGCACC
CAGTCCGCCCTGAGCAAAGACCCCAACGAGAAGCGCGATCACATGGTCCTGCTGGA
GTTCGTGACCGCCGCCGGGATCACTCTCGGCATGGACGAGCTGTACAAGTAG
