# Supplementary material for: Light-gated integrator for highlighting kinase activity in living cells
Source: Nat Commun. 2024 Sep 6;15:7804. doi: 10.1038/s41467-024-51270-4 (PMC11379911; doi:10.1038/s41467-024-51270-4)
Supplement: Supplementary file 2 — Reporting Summary [file 41467_2024_51270_MOESM2_ESM.pdf]

nature portfolio

Corresponding author(s): Jin Zhang, Wei Lin

Last updated by author(s): Jul 23, 2024

Reporting Summary

Nature Portfolio wishes to improve the reproducibility of the work that we publish. This form provides structure for consistency and transparency in reporting. For further information on Nature Portfolio policies, see our Editorial Policies and the Editorial Policy Checklist.

Statistics

For all statistical analyses, confirm that the following items are present in the figure legend, table legend, main text, or Methods section.

n/a	Confirmed	
☐	☒	The exact sample size (*n*) for each experimental group/condition, given as a discrete number and unit of measurement
☐	☒	A statement on whether measurements were taken from distinct samples or whether the same sample was measured repeatedly
☐	☒	The statistical test(s) used AND whether they are one- or two-sided<br>*Only common tests should be described solely by name; describe more complex techniques in the Methods section.*
☒	☐	A description of all covariates tested
☐	☒	A description of any assumptions or corrections, such as tests of normality and adjustment for multiple comparisons
☐	☒	A full description of the statistical parameters including central tendency (e.g. means) or other basic estimates (e.g. regression coefficient) AND variation (e.g. standard deviation) or associated estimates of uncertainty (e.g. confidence intervals)
☐	☒	For null hypothesis testing, the test statistic (e.g. *F*, *t*, *r*) with confidence intervals, effect sizes, degrees of freedom and *P* value noted<br>*Give P values as exact values whenever suitable.*
☒	☐	For Bayesian analysis, information on the choice of priors and Markov chain Monte Carlo settings
☒	☐	For hierarchical and complex designs, identification of the appropriate level for tests and full reporting of outcomes
☒	☐	Estimates of effect sizes (e.g. Cohen's *d*, Pearson's *r*), indicating how they were calculated

Our web collection on statistics for biologists contains articles on many of the points above.

Software and code

Policy information about availability of computer code

Data collection	Live-cell imaging data were collected using METAFLUOR 7.7 or MATscope (available on Github). 3D spheroid imaging data were collected using Leica software LAS X. Plate-reader data were collected using SparkControl Magellan 1.2.20. Flow cytometry data were collected using SONY Cell Sorter Software 2.1.6
Data analysis	Raw imaging data were analyzed using METAFLUOR 7.7 or Cell Profiler as described in the Methods. Plate-reader data were analyzed using Microsoft Excel. Flow cytometry data were analyzed using FlowJo v10. Statistical analyses were performed using GraphPad Prism 8. Raw RNA-seq data were analyzed using the web-based platform Galaxy and open-source R software 4.1.0. Differentially expressed genes were analyzed using Homer 4.11 or ShinyGO 0.77.

For manuscripts utilizing custom algorithms or software that are central to the research but not yet described in published literature, software must be made available to editors and reviewers. We strongly encourage code deposition in a community repository (e.g. GitHub). See the Nature Portfolio guidelines for submitting code & software for further information.

Data

Policy information about availability of data

All manuscripts must include a data availability statement. This statement should provide the following information, where applicable:

- Accession codes, unique identifiers, or web links for publicly available datasets
- A description of any restrictions on data availability
- For clinical datasets or third party data, please ensure that the statement adheres to our policy

The RNA sequencing data generated in this study have been deposited in the Gene Expression Omnibus (GEO) database under accession code GSE272741. The remaining data are available within the Article, Supporting Information or Source Data files. Source data are provided with this paper.

Research involving human participants, their data, or biological material

Policy information about studies with human participants or human data. See also policy information about sex, gender (identity/presentation), and sexual orientation and race, ethnicity and racism.

Reporting on sex and gender	*Use the terms sex (biological attribute) and gender (shaped by social and cultural circumstances) carefully in order to avoid confusing both terms. Indicate if findings apply to only one sex or gender; describe whether sex and gender were considered in study design; whether sex and/or gender was determined based on self-reporting or assigned and methods used. Provide in the source data disaggregated sex and gender data, where this information has been collected, and if consent has been obtained for sharing of individual-level data; provide overall numbers in this Reporting Summary. Please state if this information has not been collected. Report sex- and gender-based analyses where performed, justify reasons for lack of sex- and gender-based analysis.*
Reporting on race, ethnicity, or other socially relevant groupings	*Please specify the socially constructed or socially relevant categorization variable(s) used in your manuscript and explain why they were used. Please note that such variables should not be used as proxies for other socially constructed/relevant variables (for example, race or ethnicity should not be used as a proxy for socioeconomic status). Provide clear definitions of the relevant terms used, how they were provided (by the participants/respondents, the researchers, or third parties), and the method(s) used to classify people into the different categories (e.g. self-report, census or administrative data, social media data, etc.) Please provide details about how you controlled for confounding variables in your analyses.*
Population characteristics	*Describe the covariate-relevant population characteristics of the human research participants (e.g. age, genotypic information, past and current diagnosis and treatment categories). If you filled out the behavioural & social sciences study design questions and have nothing to add here, write "See above."*
Recruitment	*Describe how participants were recruited. Outline any potential self-selection bias or other biases that may be present and how these are likely to impact results.*
Ethics oversight	*Identify the organization(s) that approved the study protocol.*

Note that full information on the approval of the study protocol must also be provided in the manuscript.

Field-specific reporting

Please select the one below that is the best fit for your research. If you are not sure, read the appropriate sections before making your selection.

☒ Life sciences ☐ Behavioural & social sciences ☐ Ecological, evolutionary & environmental sciences

For a reference copy of the document with all sections, see nature.com/documents/nr-reporting-summary-flat.pdf

Life sciences study design

All studies must disclose on these points even when the disclosure is negative.

Sample size	No sample-size calculations were performed. Sample sizes were similar to those generally employed in the field.
Data exclusions	Cells that died or appeared unhealthy during the course of live cell imaging were excluded from analysis. Cells that did not exhibit a stable baseline intensity were excluded from analysis. All exclusion criteria were predetermined.
Replication	Unless otherwise noted, all experiments were repeated at least 3 times. All replication attempts were successful, and number of cells and replicates are indicated in figure legends.
Randomization	Cells were randomly seeded into experimental groups after resuspension.
Blinding	Blinding was not performed as it was not necessary. Objective quantitative data was generated and analyzed, and no subjective classifications were performed.

Reporting for specific materials, systems and methods

We require information from authors about some types of materials, experimental systems and methods used in many studies. Here, indicate whether each material, system or method listed is relevant to your study. If you are not sure if a list item applies to your research, read the appropriate section before selecting a response.

Materials & experimental systems

n/a	Involved in the study
☐	☒ Antibodies
☐	☒ Eukaryotic cell lines
☒	☐ Palaeontology and archaeology
☒	☐ Animals and other organisms
☒	☐ Clinical data
☒	☐ Dual use research of concern
☒	☐ Plants

Methods

n/a	Involved in the study
☒	☐ ChIP-seq
☐	☒ Flow cytometry
☒	☐ MRI-based neuroimaging

Antibodies

Antibodies used	For western blotting, primary antibodies:<br>Cell Signaling Technologies (Denvers, MA USA) were used at 1:1000 dilution unless otherwise noted: Myc-Tag Rabbit Antibody (2278), CREB Rabbit Antibody (#9197), Phospho-CREB (Ser133) Rabbit Antibody (#9198), p44/p42 MAPK (Erk1/2) Rabbit Antibody (#4695) and Phospho-p44/42 MAPK (Erk1/2, Thr202/Tyr204) Mouse Antibody (#9106)<br>For western blotting, secondary antibodies:<br>The horseradish peroxidase-labeled goat anti-rabbit (PI31460) and the horseradish peroxidase-labeled goat anti-mouse (PI31430) were purchased from Pierce.
Validation	All the antibodies used in the study are common, well-established commercially available antibodies. All antibodies have been validated by the manufacture.

Eukaryotic cell lines

Policy information about cell lines and Sex and Gender in Research

Cell line source(s)	HEK293T, HeLa and MCF-7 cells were used in this study. All cell lines were obtained from ATCC.
Authentication	Cell line identities were verified via STR analysis by the commercial source and then maintained separately and isolated from one another to avoid cross-contamination.
Mycoplasma contamination	All cell lines were determined to be free of mycoplasma contamination based on weekly DNA staining.
Commonly misidentified lines (See ICLAC register)	No misidentified cell lines were used in this study.

Plants

Seed stocks	*Report on the source of all seed stocks or other plant material used. If applicable, state the seed stock centre and catalogue number. If plant specimens were collected from the field, describe the collection location, date and sampling procedures.*
Novel plant genotypes	*Describe the methods by which all novel plant genotypes were produced. This includes those generated by transgenic approaches, gene editing, chemical/radiation-based mutagenesis and hybridization. For transgenic lines, describe the transformation method, the number of independent lines analyzed and the generation upon which experiments were performed. For gene-edited lines, describe the editor used, the endogenous sequence targeted for editing, the targeting guide RNA sequence (if applicable) and how the editor was applied.*
Authentication	*Describe any authentication procedures for each seed stock used or novel genotype generated. Describe any experiments used to assess the effect of a mutation and, where applicable, how potential secondary effects (e.g. second site T-DNA insertions, mosiacism, off-target gene editing) were examined.*

Flow Cytometry

Plots

Confirm that:

☒ The axis labels state the marker and fluorochrome used (e.g. CD4-FITC).

☒ The axis scales are clearly visible. Include numbers along axes only for bottom left plot of group (a 'group' is an analysis of identical markers).

☒ All plots are contour plots with outliers or pseudocolor plots.

☒ A numerical value for number of cells or percentage (with statistics) is provided.

Methodology

Sample preparation	HEK293T cells were collected using 0.25% trypsin and washed once with 1×DPBS. Cells were then resuspended in HBSS buffer containing 1% FBS and strained (FALCON) to prevent clogging.
Instrument	SONY SH800S Cell Sorter
Software	Flow cytometry data were collected using SONY Cell Sorter Software 2.1.6 and analyzed using FlowJo v10
Cell population abundance	The final sorted population was around 30% of the total events.
Gating strategy	Gating strategy in Supplementary Figure 8: Cell events were first separated from debris using gate A (forward scatter vs. side scatter). Single cells were then separated from aggregates using gate B (forward scatter width vs. forward scatter). mCherry+ cells were separated using gate C, and H2B-EGFP+ cells were then displayed by histogram (panel c).<br>Gating strategy in Supplementary Figure 17 and 18: Cell events were first separated from debris using gate A (forward scatter vs. side scatter). Single cells were then separated from aggregates using gate B (forward scatter width vs. forward scatter). mCherry+ cells were separated using gate C and mTagBFP2-GαsR201C+ cells were then separated using gate F. PKI-EGFP+ or EGFP+ cells were sorted using gate I-1.

☒ Tick this box to confirm that a figure exemplifying the gating strategy is provided in the Supplementary Information.
