# Supplementary material for: Light-gated integrator for highlighting kinase activity in living cells
Source: Nat Commun. 2024 Sep 6;15:7804. doi: 10.1038/s41467-024-51270-4 (PMC11379911; doi:10.1038/s41467-024-51270-4)
Supplement: Supplementary file 3 — Description of Additional Supplementary Files [file 41467_2024_51270_MOESM3_ESM.pdf]

Supplementary Data 1

11 potential PKA inhibitors.

Supplementary Data 2

14 potential PKA activators.

Supplementary Data 3

1064 DEGs from A-KINACT effector cells.

Supplementary Data 4

410 DEGs from A-KINACT control cells.

Supplementary Data 5

GO analysis of 106 PKA upregulated DEGs.

Supplementary Data 6

GO analysis of 731 ERK upregulated DEGs.
